# Review and future outlook for the removal of microplastics by physical, biological and chemical methods in water bodies and wastewaters

**DOI:** 10.1007/s10661-025-13883-0

**Published:** 2025-03-19

**Authors:** Marco Antonio Alvarez Amparán, Adriana Palacios, German Miranda Flores, Pedro Manuel Castro Olivera

**Affiliations:** 1 Departamento de Ingeniería Química, Facultad de Química, Universidad Nacional Autónoma de México, Ciudad de Mexico, 04510 México; 2 Departamento de Ingeniería Química, Universidad de Las Américas Puebla, Alimentos y Ambiental. Santa Catarina Mártir, Puebla. C.P. 72810, San Andrés Cholula, México

**Keywords:** Microplastic removal, Microplastic degradation, Physical removal, Chemical degradation, Biological degradation, Microplastic review

## Abstract

Microplastics (MPs) have become a major global environmental problem due to their accelerated distribution throughout different environments. Their widespread presence is a potential threat to the ecosystems because they alter the natural interaction among their constituent elements. MPs are considered as emergent pollutants due to the huge amount existing in the environment and by the toxic effects they can cause in living beings. The removal of MPs from water bodies and wastewaters is a control strategy that needs to be implemented from the present on and strictly constantly in the near future to control and mitigate their distribution into other environments. The present work shows a detailed comparison of the current potential technologies for the remediation of the MPs pollution. That is, physical, biological, and chemical methods for the removal of MPs from water bodies and wastewaters. Focusing mainly on the discussion of the perspective on the current innovative technologies for the removal or degradation of the MPs, rather than in a deep technical discussion of the methodologies. The selected novel physical methods discussed are adsorption, ultrafiltration, dynamic membranes and flotation. The physical methods are used to modify the physical properties of the MPs particles to facilitate their removal. The biological methods for the removal of MPs are based on the use of different bacterial strains, worms, mollusks or fungus to degrade MPs particles due to the hydrocarbon chain decrease of the particles, because these kinds of microorganisms feed on these organic chains. The degradation of MPs in water bodies and wastewaters by chemical methods is focusing on coagulation, electrocoagulation, photocatalysis, and ozonation. Chemical methods achieve the degradation of MPs by the modification of the chemical structure of the particles either by the change of the surface of the particles or by attacking radicals with a high oxidation capacity. Additionally, some interesting combinations of physical, chemical, and biological methods are discussed. Finally, this work includes a critical discussion and comparison of several novel methods for the removal or degradation of MPs from water bodies and wastewaters, emphasizing the areas of opportunity and challenges to be faced.

**Graphical abstract:**

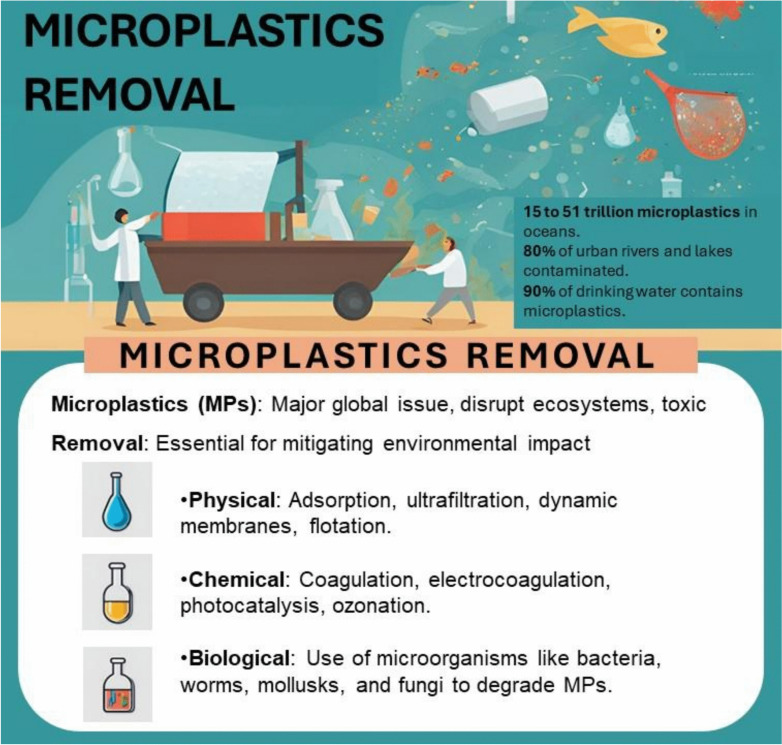

## Introduction

While plastics have been produced for a long time, concern about the presence of plastics and microplastics (MPs) in water bodies has only been studied for less than 50 years. MPs are plastic particles whose longest dimension is less than 5 mm (Ramasamy et al., 2021). MPs have become an increasing concern worldwide due to the substantial amount of solid waste and by their negative impact on the environment and living beings. Consequently, MPs are classified as micropollutants and emerging pollutants. The adverse effects of MPs as emerging pollutants have been documented extensively in published literature. The presence of MPs has been linked to liver and gut diseases, various disruptions of the nervous system such as oxidative stress and neurotoxicity, as well as reproductive problems or cancer (Wu et al., 2021; Blackburn & Green, 2022). However, more detailed studies regarding the impact of MPs on human health should be conducted.

According to the (Walker & Fequet, 2023), global plastic production has increased nearly 230-fold from 1950 to 2019, reaching 368 million metric tons in 2019. In this sense, (Stegmann et al., 2022) was pointing to a doubling projection of 750 million metric tons of MPs by 2050. The proliferation of plastic waste has intensified due to the non-degradability of most plastics under environmental conditions, with over 33% of the produced plastic designated for single-use or non-reusable products. According to the European Parliament a significant proportion of plastic waste ends up in landfills and, ultimately, in the environment (Da Costa et al., 2020). Instead of undergoing degradation, most plastics gradually fragment into smaller particles, commonly referred to as MPs and nanoplastics (NPs). These particles, whether in the form of larger or smaller plastics, entail profound and detrimental consequences for ecosystems, biota, and the environment.

Ongoing investigations into the potential impacts of MPs on human health, as suggested by Campanale et al., 2020, indicate adverse effects on the immune system, hormonal balance, and various facets of human wellbeing. The pathway through which MPs enter the human body, via the consumption of contaminated water or food, emphasizes the urgency for intensified research in this pivotal area. Beyond human health, the ramifications extend to the tourism and recreation industries, where water bodies laden with MPs experience a diminishing aesthetic appeal and recreational value, potentially culminating in economic impacts for businesses reliant on clean water (Amobonye et al., 2021). Furthermore, industrial processes, such as textile production and manufacturing, contribute to the release of MPs into water, exacerbating the detrimental effects on the environment and public health, consequently posing a risk of reputational damage for associated companies (Li et al., 2018a, 2018b; Sana et al., 2020).

The escalating accumulation of MPs and NPs in both terrestrial and aquatic ecosystems raise substantial concerns, with concentrations reaching up to 1.9 million particles per square meter in some terrestrial environments and up to 10,000 particles per cubic meter in aquatic systems (Kiran et al., 2022; Ratnasari et al., 2024). Given their adverse effects on the functions of ecosystems and human health, the adoption of sustainable plastic waste management techniques becomes imperative in the pursuit of the United Nations Sustainable Development Goals. The alarming situation is compounded by the adverse impacts of widely distributed MPs and NPs in the ecosystem, originating from direct contamination by personal care products, synthetic fabrics, and various industries.

The behavior and fate of MPs in the environment are significantly influenced by material characteristics. The density of plastics determines their initial residence in the water column, with lower-density plastics being more likely to float near the surface. The crystallinity of the MPs affects the ease of oxidative degradation and fragmentation during weathering, with highly crystalline plastics exhibiting greater resistance. Oxidation resistance is determined by the chemical structure, affecting the susceptibility of oxidative degradation and subsequent fragmentation. Biodegradability affects the rate of mineralization and potential removal of plastics from the water column or sediment. Residual monomers and additives can leach from MPs, potentially impacting marine organisms through toxicity and bioavailability. Additionally, surface properties influence fouling rates, which, in turn, affect the weathering and sinking of MPs (Ratnasari et al., 2024; Andrady, 2017; Blackburn & Green, 2022; Zhang et al., 2020; Kuan-Ju et al., 2021).

The criteria for visually sorting of plastics include size-based categories such as macroplastics, mesoplastics, plastics, MPs, mini-microplastics, and NPs (Crawford & Quinn, 2017; Windsor et al., 2019). These criteria aid in the classification and characterization of MPs in environmental samples. Different shapes of MPs, including pellets, microbeads, fragments, fibers, films, and foam, interact uniquely with the environment, affecting their transport, bioavailability, and interactions with organisms. The adverse effects of MPs encompass bioaccumulation, pollutant transportation, animal ingestion, and human health risks, highlighting the need for comprehensive strategies to address MP pollution. Understanding the sources of MPs is crucial for effective mitigation (Crawford & Quinn, 2017).

Table 1 shows how MPs can be categorized based on their physical–chemical properties, the particle size, the source, the shape and their effects on the environment. Some examples of physical–chemical properties of the MPs are density, crystallinity, oxidation resistance or biodegradability. The particle size determines the method of detection or visualization in water samples. The source of the MPs determines the form of elimination thereof. The shape will help to select an adequate method to separate and eliminate MPs. The effects of the MPs imply their impact on the environment or on human health.

**Table 1:** Criteria for the characterization of plastics as a function of material properties, size, source, shape, and effects

	Characteristic	Influence on the behavior of MPs	
Material characteristics of plastics influencing the behavior of their microparticles Andrady, 2017	Density	Buoyancy in seawater determines where in the water column the MPs are most likely to initially reside	
	Partial crystallinity	The degree of crystallinity determines the ease of oxidative degradation and fragmentation during weathering	
	Oxidation resistance or weatherability	Chemical structures determine how easily oxidizable plastic will be in the environment. Fragmentation is a consequence of extensive oxidative degradation	
	Biodegradability	Determines the rate of mineralization and potential partial removal of plastics from the water column or sediment	
	Residual monomer	Toxicity of leaching residual monomers from MPs to marine organisms that ingest plastics	
	Transport properties	Bioavailability of residual monomers and additives absorbed by the MPs depends on their leaching rates in the environment	
	Additives	Concentration and toxicity of additives in MPs may contribute to the adverse impacts on species that ingest them	
	Surface properties	Rate of fouling of floating debris determines rates of weathering and sinking of MPs	
Criteria for the visual sorting of MPs Crawford & Quinn, 2017; Windsor et al., 2019	Type	Size range	Visualization/Technique
	Macroplastics	≥ 25 mm	Naked eye—Visual counting
	Mesoplastics	< 25 mm to 5 mm	Naked eye or optical microscope—Neuston nets or sieving
	Plastic	< 5 mm	-
	Microplastics	< 5 mm to 1 mm	Optical microscope—Microfilters < 1 μm separation
	Mini-microplastics	< 1 mm to 1 mm	-
	Nanoplastics	< 1 mm	Electron microscope—Nanofilters
By source Hamidian et al., 2021	Category	Description	Examples
	Primary	Plastics that were produced on a micro-scale	Cosmetic products, microfibers, microbeads
	Secondary	Breakdown of bigger plastic objects	Photochemical, biological, and/or mechanical degraded plastic
By shape Crawford & Quinn, 2017	Category	Size	Definition
	Pellet	< 5 mm to 1 mm	Small spherical piece of plastic
	Microbead	< 1 mm to 1 mm	Small spherical piece of plastic
	Fragment	< 5 mm to 1 mm	Irregularly-shaped piece of plastic
	Microfragment	< 1 mm to 1 mm	Irregularly-shaped piece of plastic
	Fiber	< 5 mm to 1 mm	Strand or filament of plastic
	Microfiber	< 1 mm to 1 mm	Strand or filament of plastic
	Film	< 5 mm to 1 mm	Thin sheet or membrane-like piece of plastic
	Microfilm	< 1 mm to 1 mm	Thin sheet or membrane-like piece of plastic
	Foam	< 5 mm—1 mm	A piece of sponge, FM, or FM-like plastic material
	Microfoam	< 1 mm—1 mm	A piece of sponge, FM, or FM-like plastic material
By effect Lwanga et al., 2017a, 2017b	Category	Effect	Description
	Environmental	Bioaccumulation	Concentration of MPs increases as organisms are eaten by others
		Pollutant transportation	Heavy metals and other toxic substances are attached to the MPs and are transported through water
		Animal ingestion	Obstruction of respiratory and/or digestive tracts, leading to death by starvation or lack of oxygen
	Human	Ingestion	The presence of MPs in the human body can increase the risk of infertility, exposure to toxic substances, and lung diseases

As shown in Table 1, there are multiple methods to detect MPs. These are of utmost importance because MPs have different sizes, shapes, sources, and interactions with the environment. Therefore, based on the correct detection of MPs, the selection of appropriate removal/degradation methods can be made for the elimination of these micropollutants from aqueous environments. Thus, the separation and elimination of MPs will be selected based on the environment in which they are found and the properties of these MPs to obtain the highest possible removal efficiency.

Table 2 provides a comprehensive summary of selected publications (from 2016 to 2023) that have reported the presence of MPs in various water bodies across different geographical regions. The information in this compilation is categorized based on the geographic location, the water body under investigation, the type of MPs detected, the concentrations of MPs observed, the specific particle shape of MPs, and the MPs size. The studies featured in Table 2 reveal the widespread occurrence of MPs in diverse aquatic environments, including lakes, seas and rivers. Therefore, underscoring the significant global challenge posed by the presence of these particles and the need for effective strategies for their removal or degradation.

**Table 2:** Summary of recent research on MPs in different regions and water bodies ^*^

Place of research	Water body	Concentration	Type of MP	Particle size	Reference
Agadir metropolis (Moroccan Atlantic)	Influent and affluent of Aourir and M'zar wastewater treatment plants	519 MPs/L in Aourir and 86 MPs/L in M'zar	PE, PP, and PS	100 – 500 μm	Bayo et al., 2023
Bahía Blanca Estuary (BBE), Argentina	Estuary	3.69 ± 2.02 MPs/g in middle zone and 3.54 ± 3.52 MPs/g in external zone	PET, PL, cotton, polyamide, cellulose	0.5 – 5 mm	Colombo et al., 2023
Beyhan dam lake; Elazığ, Turkey	Lake	2.55 ± 2.80 MPs/g	PP (53.3%), copolymer ethyl-acrylate (33.3%) and polychloroprene (13.3%)	1001 – 2000 μm	Atamanalp et al., 2023
Southern coast of Apulia in southern Italy	Salento peninsula	5.4 items/individual in S. pilchardus and 4.6 items/individual in B. boops	PE (fragments (FRs), filaments, or pellets (PT)), PP (FRs or Films (FI)), PS, and PET (FRs and filaments)	> 500 μm for FR, FI and PT and > 1 mm for fibers (FB)	Trani et al., 2023
Length of the Rhine River; Rotterdam, Netherlands; Basel, Switzerland	Rhine river	892,777 particles/km^2^—3.9 million particles/km^2^	PS (29.7%), PP (16.9%), acrylate (9.3%), PL (5.1%), polyvinyl chloride (PVC) (1.7%) and other types (13.6%)	400 – 900 μm	Mani et al., 2016
Anhui, Hubei, Hunan, Jiangsu, Jiangxi, Qinghai, Sichuan and Yunna provinces, China	Yangze river	1.27 items/L, 286.20 items/L, and 338.09 items/L	FB are dominant in all compartments and account for more than 40% in all sampling sites	-	Yuan et al., 2022a, 2022b
Hunan Province to the north, into Dongting Lake and the Yangtze River	Xiangjiang river, China	From 144 to 510 items/kg with an average abundance at 288 ± 60 items/kg	-	-	Yin et al., 2022
Taghazout coast, central Atlantic part of Morocco	Urbanized beach	915 MPs/kg (2018)—1448 MPs/kg (2019)	-	-	Ben-Haddad et al., 2022
Sanggou Bay, China	Marine environment	20.06 ± 4.73 items/L	PE	< 0.5 mm	Xia et al., 2021
Selengor, Malaysia	Klang river	0.50—1.75 particles/g	PE-PP-diene and PL as fibers (91%)	30—1850 μm,	Zaki et al., 2021
Sari, Iran	Water treatment plant	380 ± 52.5 – 423 ± 44.9 MPs/m^3^	PL and PE as fibers and particles	< 300 μm and ≥ 500 μm	Razeghi et al., 2021
Oaxaca, Mexico	Water in rainforest, pine plantation, savanna, and pasture soils	1.49 and 1.53 particles/g	-	150—500 μm	(Álvarez-Lopeztello et.al., 2021)
Alberta, Canada	River	4.6—88.3 particles/m^3^	PE or PP as FRs	> 1 mm, 1 mm – 500 μm, 500 – 250 μm, 250 – 125 μm, and 125 – 53 μm	Bujaczek et al., 2021
Hengchun Peninsula, Taiwan	Coastal waters	-	Fiber	-	Sun et al., 2021
(Mallorca, Barcelona, Malaga, among others) Mediterranean coastal countries Spain, Italy, France, Croatia, and others	Mediterranean Sea	45.9 ± 23.9 MPs/kg (Palma de Mallorca); 280.3 ± 164.9 MPs/kg (Málaga); 113.2 ± 88.9 MPs/kg (Beach sediment-Denia and Barcelona)	FB, PE	< 0.5 mm, 0.5 – 5 mm)	Fytianos et al., 2021
USA, Canada	Lake	65.2 particles/kg (Lake Michigan); 431 particles/kg (Lake Erie)	PET, high-density polyethylene (HDPE), semisynthetic cellulose (S.S. Cellulose), PP, and PVC	0.1250 − 0.3549 mm	Lenaker et al., 2021
(Nansha, Guangdong Province) China	Aquaculture system (near the Pearl River)	6.6 × 10^3^ – 263.6 × 10^3^ items/m^3^ (surface water) and 566.67 – 2500 items/kg (sediment)	Cellulose, PP, and PE	< 1 mm	Li et al., 2021a
Guangdong Province, China	Coast	850 to 3500 items/L (surface water) and 433.3 to 4166.3 items/kg (sediment)	Rayon (38.2%), PET (16.4%), and ethylene/vinyl acetate copolymer (12.7%)	< 0.5 mm, 0.5–1 mm, 1–2 mm, 2–3 mm, 3–4 mm, and 4–5 mm	Li et al., 2021b
Iceland	Sea	0.119—0.768/g	FB	> 250 μm − 5 mm	Loughlin et al., 2021
Aosta Valley, Italy	Snow	0.39 ± 0.39 MPs/L and 4.91 ± 2.48 MPs/L	fibers, FRs. PE, PET, HDPE, PL, LDPE, PP and PU	50—1910 µm	Parolini et al., 2021
Portugal	Sea	0.30 ± 0.63 MPs/ind (S. plana); 2.46 ± 4.12 MPs/ind (S. colias)	PL and PE	73 μm – 4680 μm	Pequeno et al., 2021
Japan, Okinawa	Sea	-	LDPE	(2.53 ± 0.85) μm and 20 μm	Ripken et al., 2021
Baltic sea	Sea	237 ± 277 ng/m^3^; 106 ± 209 ng/m^3^	PE, PP, and PET	73 ± 45 mm;154 mm and 23 mm	Uurasjärvi et al., 2021
Michigan, Ontario, around Great Lakes	Lake	52,000 particles/km^2^, 55,700 particles/km^2^ and 5,400 particles/km^2^	MFB	-	Earn et al., 2021
Ontario, Canada Lake Simcoe	Lake	0—0.7 particles/L (surface water); 0.4–1.3 particles/m^3^ (manta trawl), and 8.3–1070 particles/kg (sediment samples)	PE and PP	> 125 mm	Felismino et al., 2021

*see Abbreviation section, page 1

As shown in Table 2, many plastics with several particle sizes and concentrations are present in multiple locations and water bodies from different regions throughout the world. This demonstrates the importance of MP removal, since the contamination has covered extensive dimensions. In addition, as shown in Table 1, the MPs present both in the environment and in the human body have serious consequences that can affect vital functions of the biological cycles.

The objective of this review paper is to discuss and compare different water treatment methods focused on the removal of microplastics from water bodies, which has been an emerging problem in recent times. Many organizations, companies and several sectors do not focus their resources on plastic removal because there is no strict government regulation that prioritizes their removal. This fact triggers environmental and health problems that irreversibly damage the ecosystems and to society. Since many recent publications show the effect of microplastics on living beings rather than the possible alternatives for their removal or degradation, in this review we want to demonstrate the alternatives that currently exist for the removal of microplastics by physical, chemical and biological methods to mitigate their environmental impact and consequently improve the quality of life of human beings. Therefore, an overview and outlook of novel methodologies for the removal of microplastics from water bodies will be addressed and discussed.

The methodologies were grouped in three categories for discussion: methodologies for the removal of MPs by physical, biological and chemical treatments. A description of some selected and representative works in each category will be made in order to highlight the impact of each, to highlight the novelty of technology for the elimination of MPs rather than to delve into its fundamentals and principles. Finally, an analysis of the technologies as a whole and a perspective for the removal or elimination of MPs will be made.

## Treatment methods for the removal of microplastics

When MPs reach an aquatic environment, they may be ingested by different animal species, and depending on the size of the MPs, these might reach humans when consumed. The problem of the biological impact of MPs ingestion has been well identified and focuses both on the physical damage on animal species or humans by the blockage that these micropollutants may cause on the digestive tract of the animal that ingests them, as well as on the leaching of the chemical components that make up the plastic, together with the contaminants that may have been adsorbed on the particles (Davtalab et al., 2023; Fu et al., 2021).

The problem appears at wastewater treatment plants (WWTPs), since their aim is not focused on the removal of MPs and NPs. However, due to the large volumes of wastewater treated in WWTPs, and depending on the technology implemented at each, a greater number of plastic particles and some types of MPs could be retained. Nowadays, there are considerable sources of MPs, and there is currently no certainty that all the MPs can be removed from the influents that reach the WWTPs (Bakaraki et al., 2021). Also, it is worth mentioning that the techniques currently used to treat wastewater are not designed to retain minute materials, since the original designs were thought to treat other types of pollutants such as biological agents, heavy metals, organic and inorganic compounds, among some others (Zhou et al., 2019).

The following section will mention the novel physical, biological, and chemical methods for MPs removal. The principle operating conditions and efficiency of MPs removal will be discussed in detail.

### Physical methods

**Fig. 1:**
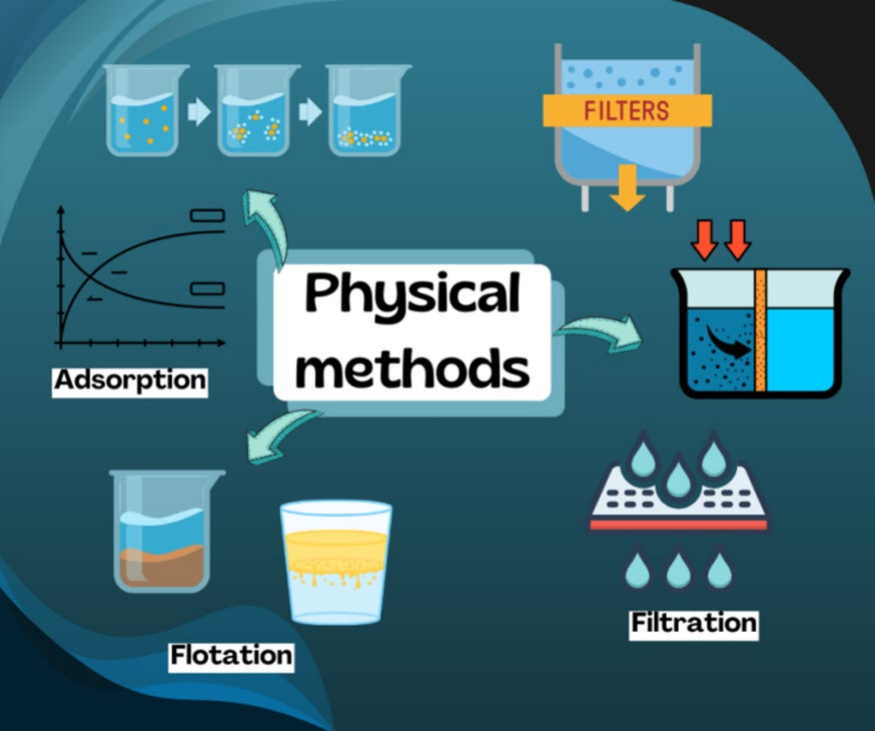
Physical methods for water treatment

Physical methods for the removal of MPs are based on the change of the physical properties of the MPs particles to facilitate their removal from the water body. Specific density, particle size, or particle volume are common parameters or properties to be modified for a better performance of physical processes such as adsorption, filtration with membranes, or flotation (Bahuguna et al., 2021). Figure 1 shows the water treatment methods addressed in this section: adsorption, filtration (ultrafiltration and dynamic membranes) and flotation.

Physical methods for MPs removal essentially rely on the alteration of the physical properties of the MPs particles to facilitate their separation from water by processes such as adsorption, filtration and flotation, see Fig. 1. The particle size, density and volume are the main parameters modified on the above commented physical processes to improve the performance of the MP removal. These physical methods for the MPs removal are effective. However, the efficiency of these ones is limited by the nature of the adsorption materials and the capacity of the filtration and flotation processes involved. Therefore, this review shows the differences in the efficiency reported by using several materials in adsorption, ultrafiltration, dynamic membranes, as well as for the use of several technologies used for flotation. Finally, an outlook about their feasibility in large-scale applications for the MP removal is made.

#### Adsorption

**Fig. 2:**
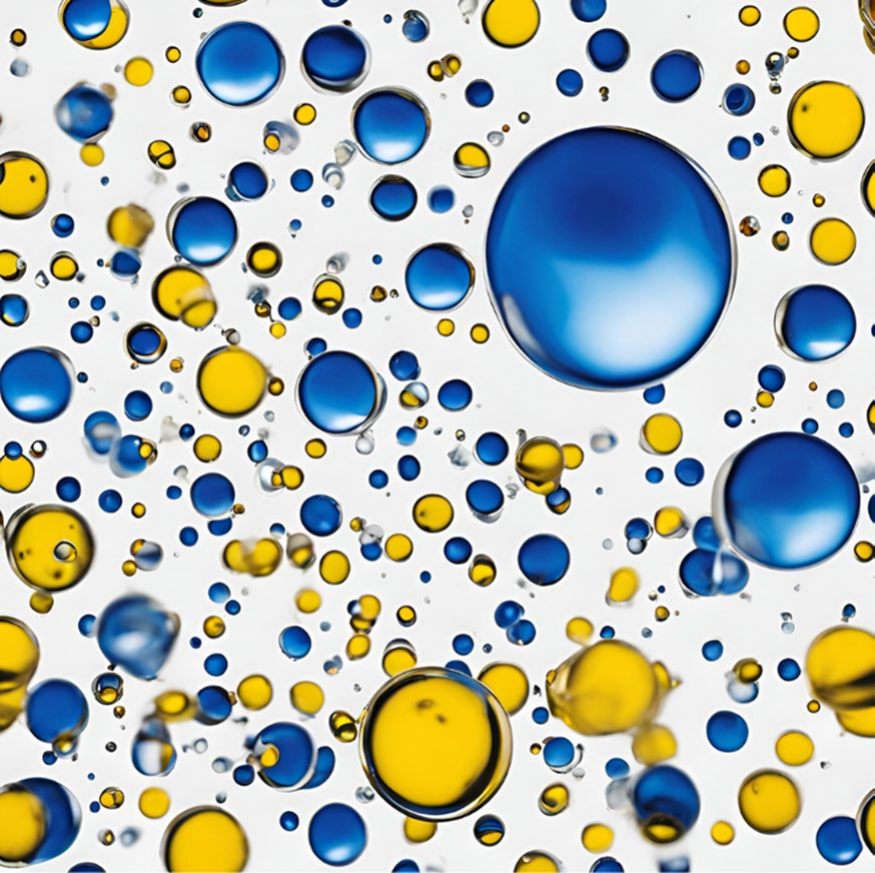
Schematic representation of MPs contained in water

Adsorption as physicochemical operation refers to the removal of a solute contained in a liquid solution. However, for MPs removal adsorption refers to the retention of MPs particles (adsorbate) on the surface of a solid material (adsorbent), that is a liquid–solid separation. Figure 2 shows a schematic representation of the MPs contained in water with very varied sizes and spherical shape as a simplification. Then for adsorption processes these solid particles must be retained on the surface of an adsorbent material.

One of the most common ways to represent the adsorption equilibrium is by means of the relationship between the amount of pollutants adsorbed on the solid surface and the concentration of pollutants in the liquid phase, for a given temperature. This relationship is known as the adsorption isotherm for a given adsorbate-adsorbent system (Rashid et al., 2021). Adsorption could be a potential method for the removal of MPs from water bodies. Different adsorbent media such as graphene oxide or biomass-derived compounds such as chitin are tested for the adsorption of MPs, where the MPs removal is due to hydrogen bonds and electrostatic attractions on the solid surface. Alginate secreted by algae and microalgae or granular activated carbon are adsorbent materials capable of removing MPs from wastewater. For NPs, it has been observed that biodegradable cellulose fibers can be used as adsorbent materials (Dey et al., 2021). Because of the wide variety of adsorbents materials, it is possible to remove different sizes of MPs, as well as other contaminants that adhere to the surface of MPs as a consequence of their dynamic factors such as: composition, structure, surface properties and binding energy (Joo et al., 2021).

Adsorption has limited use in the removal of MPs compared to other methods, due mainly to the restricted adsorption capacity of the adsorbent material. However, some researchers have developed novel materials and/or adsorption processes to achieve the desired removal effect. For example, Wang et al. (2021) carried out the removal of polystyrene (PS) from a model solution in 5-h cycles at 180 rpm. Magnetic biochar adsorbents modified with Mg, Fe, and Zn were added to the sample. Without the magnetic sample the PS removal was 25.9%, while using the magnetic absorbent the removal achieved was 94.81%. In another case, Yuan et al. (2020) used a non-metallic adsorbent such as graphene to remove MPs from a model solution (250 mg MPs/L) at pH = 6, reaching up to 76.98% of MP removal.

Additionally, adsorption/catalysis-photocatalysis processes have been developed. That is, after the adsorption process pollutants or heavy metals adsorbed on MPs surface are subsequently degraded. The degradation of pollutants is a combination of MPs adsorption on the surface of the adsorbent material and followed by an oxidation process using a catalytic/photocatalytic material. Even some authors stated that pollutants are desorbed from the MPs surface, and the oxidation is carried out into the aquatic medium or the water matrix. In this sense, the adsorption capacity of MPs for several pollutants or heavy metals has been reported in several publications. For example, Muñoz (2018) evaluated the adsorption of cadmium, lead, and fuel oil on MPs in simulated marine environment conditions, and the adsorption kinetics was determined. Six types of plastics (PP, PE, PVC, PS, HDPE, LDPE PET) were tested. The plastics were exposed to a simulated marine solution 24 h after incorporating the contaminants. The solution was analyzed 7 days after being in contact with the MPs. Maximum concentrations of adsorbed contaminants were 1076.66 mg Pb/kg MPs and 785.13 mg Cd/kg MPs for PS and 2914.39 mg fuel oil/kg MPs for LDPE. In a similar sense, novel materials such as K_2_FeO_4_ and Na_2_S_2_O_8_ were used to treat MPs samples with Pb adsorbed on their surface to determine the removal of the attached metal from their surface. After 6 h, once the adsorption/desorption equilibrium was reached, the adsorbents/catalysts (K_2_FeO_4_ and Na_2_S_2_O_8_) were added to trigger a complementary oxidation process. After 4 h, Pb removals up to 60% were achieved (Ye et al., 2020).

Based on the above discussion it can be stated that adsorption, a separation technique to remove MPs from water using adsorbent materials, where some of the most investigated currently have been for example graphene oxide, chitin, or activated carbon, is limited by the capacity of the adsorbent materials. That is, smaller MPs or MPs of complex chemical composition are difficult to remove from water. In this sense, researchers are continuously looking for novel adsorbents materials to increase MPs removal efficiency. For example, some researchers have presented promising results for improved MPs removal by proposing magnetic biochar, modified graphene or advanced materials, and they have also proposed its use with for upscaling for practical application in longer terms.

#### Membrane technology

Membranes are barriers that allow the flow of water but prevent the flow of unwanted substances as suspended solids (such as MPs) or dissolved organic or inorganic matter. Membrane water treatment processes include several technologies, among which the following stand out: direct osmosis, reverse osmosis, microfiltration, ultrafiltration and nanofiltration (Linh-Thy et al., 2023). Membranes are generally classified as isotropic or anisotropic. Isotropic membranes (generally a polymeric material) are uniform in composition and their physical nature across the cross-section of the membrane is similar to microporous or film membranes. Anisotropic membranes are non-uniform over the membrane cross-section, and they typically consist of layers which vary in structure and/or chemical composition, where the main purpose is to filter materials by density in each layer (Kumar et al., 2023). In this review, only the ultrafiltration method and dynamic membranes will be explored, which by themselves indirectly encompass all types of membranes depending on the study case.

##### Ultrafiltration

**Fig. 3:**
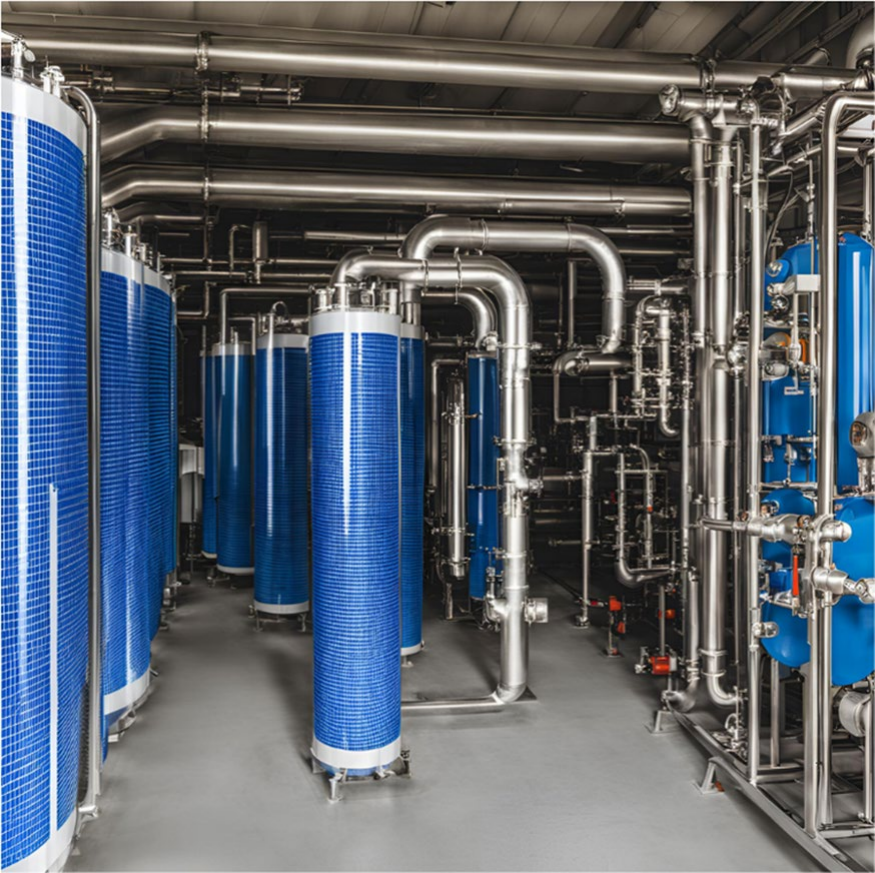
Ultrafiltration industrial process

Filtration has widely been used for the removal of particles in water, since the use of membrane technologies has been reported to have high efficiencies in the removal of MPs. The typical ultrafiltration industrial process (see Fig. 3) consists of a series or parallel configuration of several ultrafiltration units, where the rejection of one ultrafiltration unit or units is the feed of another ultrafiltration unit or units, to achieve better efficiency of MPs removal from fed water. Usually, this physical process is used when the concentration of MPs is small compared to the volumetric quantity of water and the use of chemicals is not necessary. The membrane must be replaced after it becomes saturated, however, the operation with membranes is quite economical (Singh & Hankins, 2016). Ultrafiltration is a type of filtration that is carried out using novel synthetic membranes, generally porous membranes classified by molecular cut-off weight, which is equivalent to the molecular weight of the smallest molecule that their pores can retain at 90%, and which ranges between 1000 and 500 000 g/mol, that is, molecules and macromolecules (Poerio et al., 2019). To avoid saturation or fouling due to organic and inorganic matter, the membranes are designed to a determined pore diameter in such a way that they retain only the MPs particles (Yang et al., 2021a, 2021b).

Ma et al. (2018) have reported the use of coagulants (Al and Fe) for MPs removal, since the small MPs particles (< 5 nm) cannot be retained by the membranes. The authors used a model solution of freshwater with MPs and subsequently the coagulant was added. The maximum MPs removal was 2.8%, the authors stated that this method is not as effective as other methods. Cuartucci (2020) stated that ultrafiltration is an effective method for MPs removal when the particle size has a density similar that water (0.92 to 0.97 g/cm^3^) which leads the MPs to float.

##### Dynamic membranes

During membrane filtration processes, the deposition of particles on the membrane surface, and the consequent formation of a filtration cake, is an undesirable phenomenon, since it increases mechanical resistance and, consequently, the filtration pressure. However, it has been observed that fouling can serve as a secondary membrane, sometimes defining the retention properties of the system. This phenomenon would suggest the possibility of using filter fouling as a self-forming dynamic membrane, which would allow a higher amount of retention of the solids contained in the suspension (Poerio et al., 2019).

**Fig. 4:**
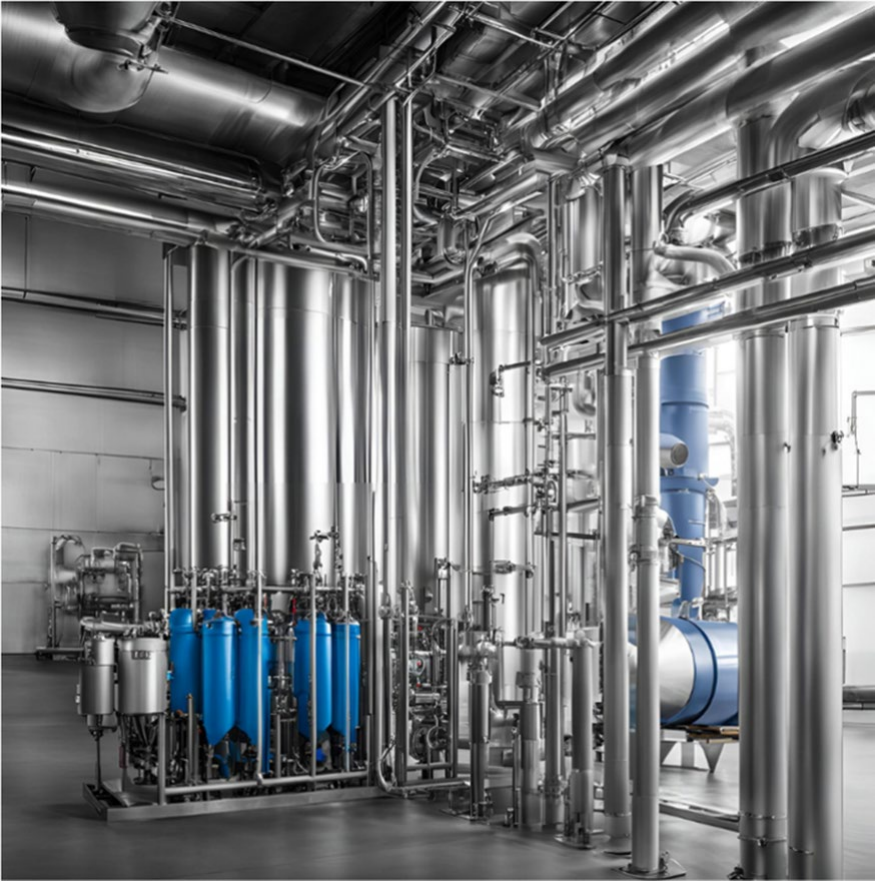
Pilot-process scheme of dynamic membranes

This technology could be suitable for separating low density and non-degradable particles, such as MPs and NPs. Dynamic membranes are a novel technology that consists of a second layer (or membrane) above or below the surface of the filtration membrane. The combination of the filtration membrane and second layer improves the filtration efficiency since one of them filters the MPs and the other could filter smaller particles or NPs. It is an interesting and competitive process since it does not involve the use of chemicals or other materials, as well as by cost-effectiveness since energy consumption is low. Dynamic membrane technology has been applied for the treatment of different effluents such as petroleum wastewater, municipal wastewater and surface water treated in WWTPs, showing good results (Zhang et al., 2020). Also, as it was stated by Sharma et al. (2021), the dynamic membranes do not require the use of a pump (or high pump power) since the trans-membrane pressure promotes the flow of water through the membranes. Furthermore, the cleaning process can be made by air or water backwashing. Figure 4 shows representation of a conceptual pilot-process scheme of dynamic membranes in a continuous or semi-continuous process. The length of the filtration unit, using dynamic membranes, may be significantly lower due to great efficiency compared with conventional filtration processes. Also, the filtration processes using dynamic membranes units maybe it would require less filtration equipment.

In this sense, Li et al., (2018a, 2018b) used a laboratory-scale dynamic membrane with a working volume of 20 L, using a 90 μm flat sheet mesh with an effective pore diameter of 100 mm for the filtration, resulting in a removal of particles larger than 90 μm of up to 90%. Other authors, such as Lares et al. (2018), combined membranes with a bioreactor with activated sludge. The authors collected samples every two weeks over three months, and the MPs removal achieved was 99.4%. Nowadays, the most recent water treatment method with dynamic membranes is “membrane bioreactor technology” which is a future-proof solution for municipal and industrial wastewater treatment, enabling the maximization of resource recovery, minimization of costs, and implementation of a circular economy perspective in operations by reducing organic loads, MPs, and other pollutants (Cai et al., 2022).

The ultrafiltration and dynamic membranes technologies represent promising methods for MPs removal, achieving highly efficient separation of particles based on their size. Membranes permit the selective filtration of MPs while allowing water to pass through freely. However, membrane fouling, operational costs, and minor effectiveness when dealing with small-sized particles are the major issues that must be tackled. It has been highlighted, in this previous discussion, that membrane processes, particularly dynamic membranes, may present an economically viable solution, but further studies are needed to enhance their scale-up and efficiency, especially for small size MPs.

#### Flotation

**Fig. 5:**
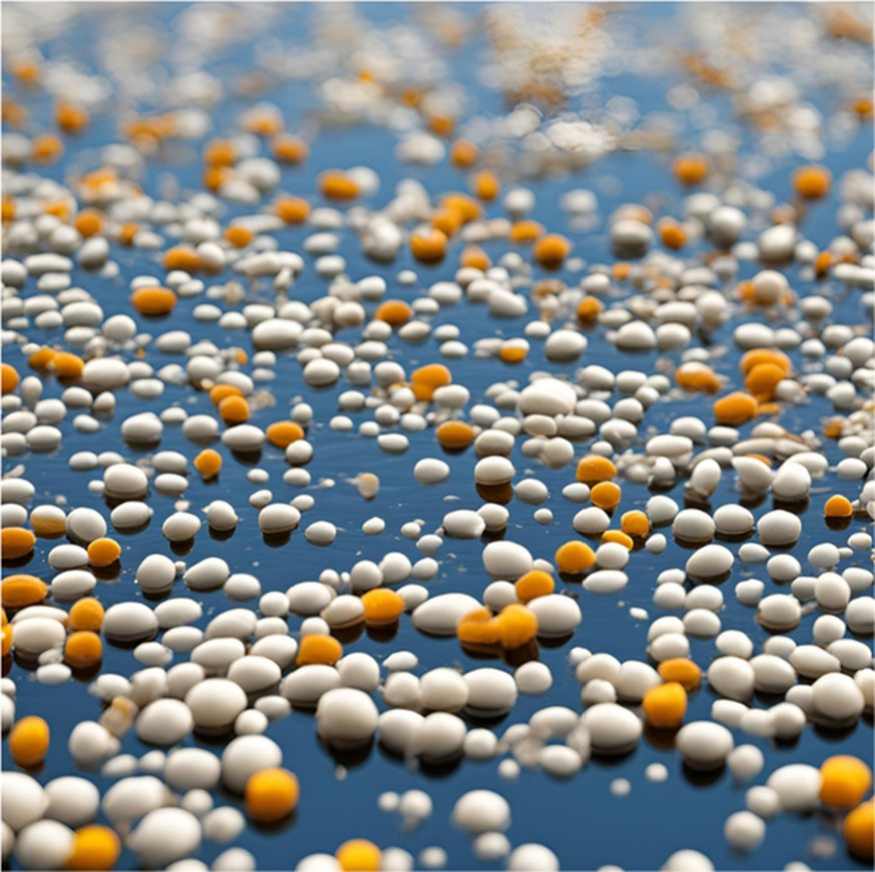
Flotation of MPs and NPs aggregates

Flotation is the process where gas (commonly air) is introduced into the water sample, creating bubbles. The generated bubbles will attach to undissolved suspended particles and therefore the volume of the bubble-particle complex increasing will gradually increase. Due to the low density of the bubbles, they tend to rise and can attach certain contaminants such as MPs and move them to the surface of the water volumes, and later the MPs could be removed by drag. Figure 5 shows an schematic representation of MPs and NPs aggregates on the surface of the water volumes, which float due to their low density, favoring its separation from the water body by means of drag or decantation. It is important to note that attachment of the bubbles to the MPs and NPs could be easier at a particular contact angle and when the bubbles are smaller (Fuerstenau & Urbina, 2018).

The most commonly flotation processes reported are mechanical flotation, dissolved air flotation and electroflotation. In the mechanical flotation process, the air bubbles are generated by a compressor and have a diameter smaller than 1 mm. For dissolved air flotation, the water is previously pressurized in an air saturation tank. Afterwards, when the pressure reached is high, the water passes through an expansion valve to a flotation tank. Finally, in the electroflotation process, gas bubbles are produced via electrolysis. That is, the hydrogen and oxygen generated by the H_2_O decomposition promote the generation of bubbles with an approximate diameter of 5 mm (Wang et al., 2020).

Mohana et al. (2021) and Pramanik et al. (2021) worked with a wastewater sample in a 2 L column at pH = 7—9 and removed MPs and NPs (PE, PVC and PES) using flotation with air bubbles. According to the authors, for PE a removal efficiency of 72% was reached, whereas for PES the best removal efficiency was 64%. Alternatively, the flotation process was coupled with a dynamic membrane process (microfiltration and ultrafiltration membranes). After the combined process, the removal efficiency for MPs and NPs was 91% and 96%, respectively. Therefore, the authors concluded that pH and the type of plastic particles affect the removal performance. In an interesting application of dissolved air flotation, Zhang et al. (2021) reported the treatment of wastewater samples to remove MPs, where the authors obtained 95% MP removal. However, according to the authors, when the dissolved air flotation process was compared with the froth flotation process (bubbles generated in a froth-like regimen) the removal efficiency decreased to 55%, suggesting that bubble size or other parameters should be researched in detail.

Mechanical flotation, dissolved air-assisted flotation and electro-flotation technologies allow the MPs removal by attaching suspended particles to air bubbles, carrying the MPs particles to the surface for easy removal. It has been pointed out that flotation can be very effective for MPs removal and that these techniques generally depend on factors such as bubble size and particle density. Also, combining technologies allows to enhance the MPs removal efficiency, i.e., flotation methods with membrane filtration. However, further research is required for optimizing the flotation parameters such as bubble characteristics for their integration into the large-scale water and wastewater treatment system efficiently.

### Biological methods

**Fig. 6:**
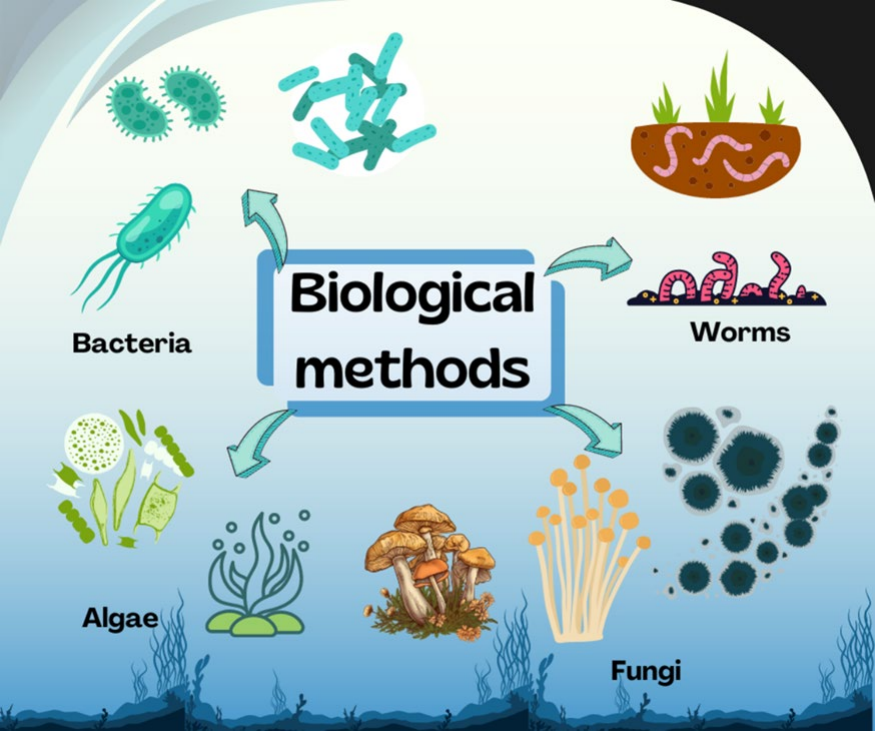
Biological agents used for MPs degradation

Commonly biological methods are used to eliminate organic load present in water bodies, in certain cases it can be used for the removal of priority pollutants. In this sense, MPs removal using biological methods has also been reported. The microorganisms present in the biological treatment feed on molecules such as weak acids, methanol, ethanol, sugars, ammonium, some salts, and even hydrocarbon chains of MPs and NPs, degrading them through their biological cycles and consequently, biomass is generated (Hossain et al., 2024). Biological treatments for plastic degradation have been studied and improved throughout the years. In recent decades, several biological alternatives have been researched for this purpose. Biological and engineering aspects are critical and must be improved to at least have a similar performance of conventional systems (Yan et al., 2023). Figure 6 shows the different organisms that can be used for the degradation of MPs through biological treatments: bacteria, worms, algae or fungus.

Microalgae are effective in purifying wastewater, as they can eliminate several substances including nutrients, heavy metals, organic and inorganic toxins and MPs particles. Microalgae accomplish degradation by utilizing sunlight, carbon dioxide (CO_2_) and other nutrients. The primary benefit of microalgae systems lies in their ability to harness solar energy within their chloroplast cells. This energy, along with CO_2_ and nutrients from wastewater, is used to produce biomass and oxygen, thereby purifying the water (Acién et al., 2016).

**Fig. 7:**
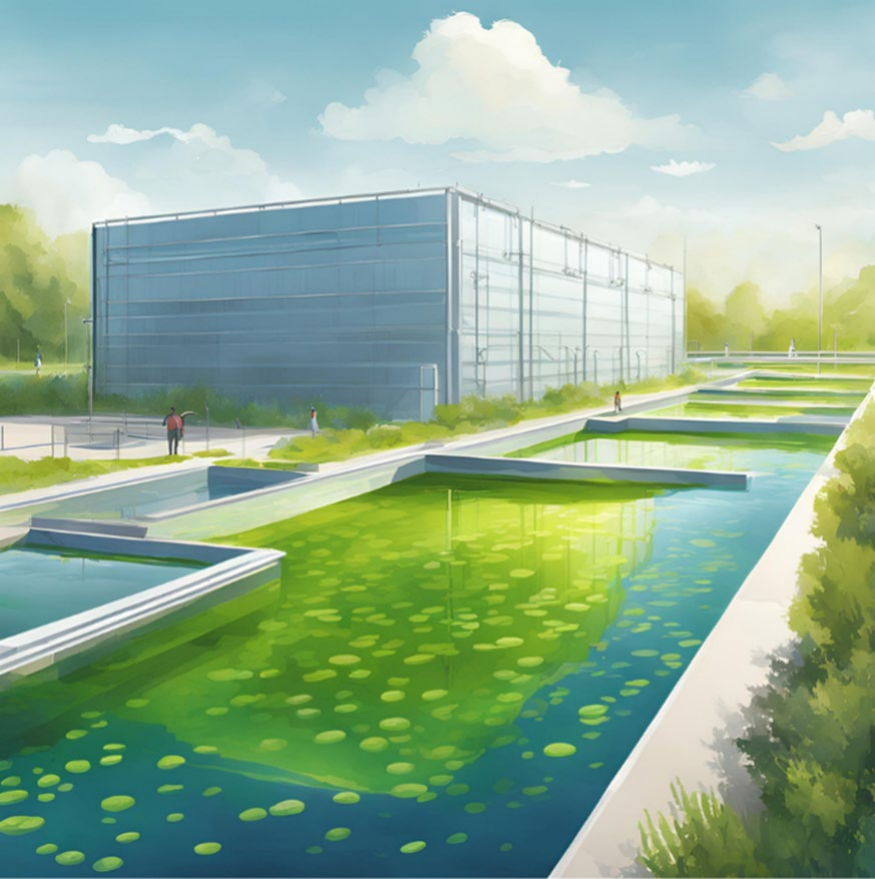
Conceptual biological MPs degradation by microalgae

MPs degradation using microalgae has been reported by Liang et al. (2024), where the authors found that the *Chlorella pyrenoidosa sp.* have the ability to accumulate and biodegrade MPs in the presence of dimethyl phthalate (DMP). Satya et al. (2023) and Chi et al. (2019) studied the species *Cylindrotheca closterium* and *Dunaliella salina*, respectively, and found that they biodegrade MPs in the presence of di-n-butyl phthalate (DBP). Zhang et al. (2016) studied *Synechocystis sp.* and *Synechococcus sp*. in the presence of DMP and observed that there was DMP degradation. However, the degradation was inhibited when the size of the MPs was greater than 20 ppm. Figure 7 shows a conceptual design for the use of microalgae cultures for the degradation of MPs from water or wastewaters volume. The treatment of a defined water or wastewaters volume implies that MPs biological degradation by microalgae is, for the moment, a semi-continuous or batch process.

MPs removal by biological methods using microalgae has shown efficient results in the purification of water and wastewaters through the removal of a wide variety of MPs, in addition to removing a wide variety of organic and inorganic toxins. For example, Chlorella pyrenoidosa, Cylindrotheca closterium, and Dunaliella salina have satisfactorily been used for the biodegradation of MPs like phthalates. Also, current research has shown that microalgae can accumulate and degrade MPs over a period of several days, but degradation is hampered when MPs exceed certain sizes. In addition to MPs removal by microalgae, greater polish of water and wastewaters could be reached using microalgae with sunlight, CO_2_, and nutrients from water and wastewaters to produce biomass, purifying the water in the process.

**Fig. 8:**
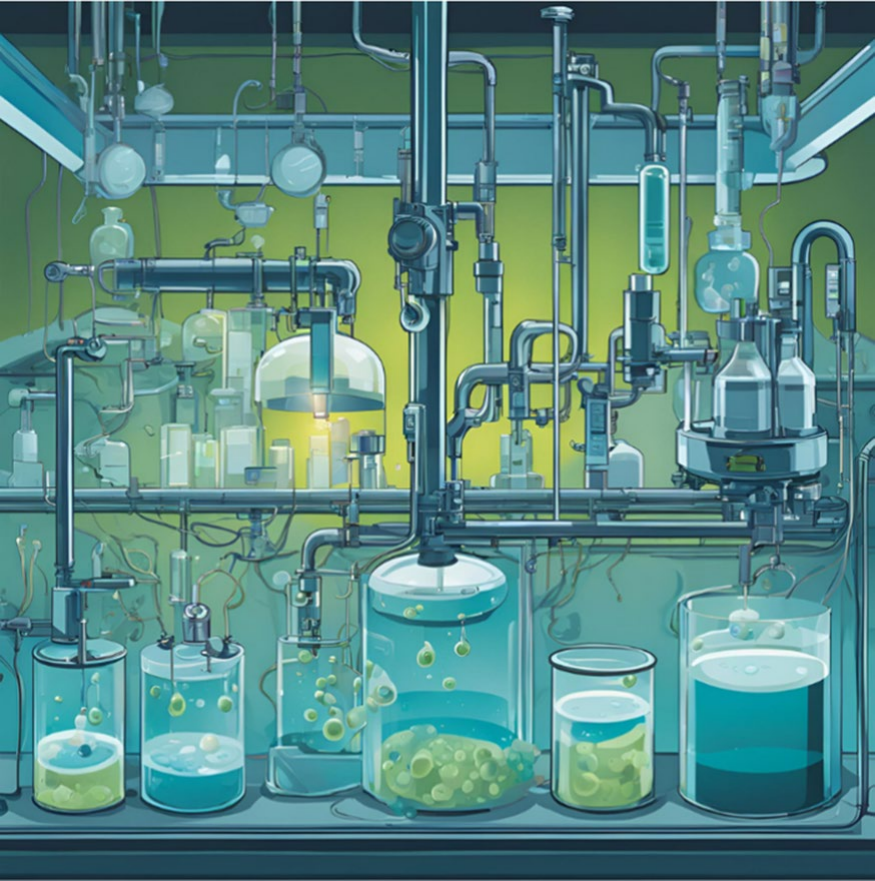
Schematic conceptual design of laboratory tests for MPs degradation by bacteria

Bacteria are an important group of microorganisms and are the most abundant of all organisms on the planet; bacteria live mainly in soil, water, and the atmosphere, and many species are well known for their ability to degrade contaminants. The main taxa with the ability to biodegrade MPs include the genera *Bacillus, Pseudomonas, Chelatococcus,* and *Lysinibacillus*. It is noteworthy that when bacteria degrade MPs, the rate of weight loss is low, typically 0 to 15%, suggesting that MPs are poorly biodegradable by these organisms (Anand et al., 2023). Figure 8 shows a conceptual design of laboratory tests for MPs degradation by bacteria. In the same sense that microalgae MPs degradation by bacteria is a semi-continuous or batch process, in the absence of the development of better technology. Various strains of microorganisms have been extensively studied for their ability to degrade plastics. For instance, the efficacy of the bacteria *Bacillus* has been studied by Yuan et al., (2022a, 2022b) and (Roberts et al., 2020) for the degradation of several MPs including PE and PET. In a similar way Auta et al. (2017) reported the degradation of PS using *Bacillus*. The bacteria type *Rhodococcus* was studied by Auta et al. (2018) and Yao et al. (2022) and it was reported PP and PE degradation.

**Fig. 9:**
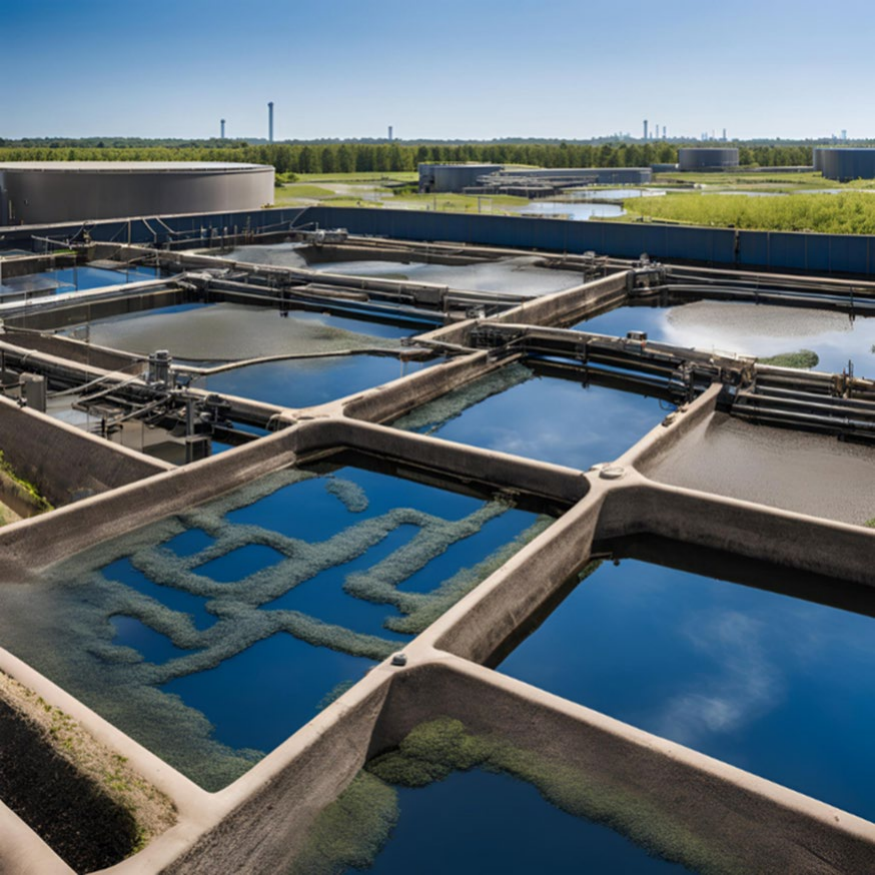
Conceptual batch design for MPs degradation by fungus or mollusks

In addition, it has reported biological MPs degradation using fungus, worms or mollusks. For example, Paco et al. (2017) found that the fungus *Zalerium maritimum* could be employed for the removal of PE. Zhang et al. (2020) has used *Aspergillus flavus* and has reported the efficiency of this fungus for the degradation of MPs in water samples. Huerta et al. (2016) and Revel et al. (2020) have reported that worms exhibit the ability to degrade MPs, including PE. Furthermore, Vosshage et al. (2018) documented the use of mollusks for the removal of MPs from water samples. Figure 9 shows a conceptual batch design for MPs degradation from water o wastewater volumes by fungus or mollusks in a WWTPs or in a space designated for that purpose.

Table 3 shows comparative data highlighting the removal percentage of different biological methods. The biological methods are categorized by kingdom, organism type (strain), and the type of MPs used in the corresponding study. For each organism, the removal is indicated for four types of plastics: PE, PET, PS and PP. The MP degradation is specified in terms of weight loss percentage (%wl) and days (d).

**Table 3:** Effect of biological organisms on the degradation of different MPs

Kingdom	Organism	PE	PET	PS	PP	Reference
Monera	*Bacillus cereus*	1.6% wl per 40d	6.6% wl per 40d	7.4% wl per 40d	-	Auta et al., 2017
	*Bacillus gottheilii*	6.2% wl per 40d	3% wl per 40d	5.8% wl per 40d	3.6% wl per 40d	Auta et al., 2017
	*Bacillus*	-	-	-	6.4% wl	Auta et al., 2018
	*Rhodococcus ruber*	0.86% wl per week	-	-		Sivan et al., 2006
	*Rhodococcus*	-	-	-	4% wl	Auta et al., 2018
	*Bacillus* + *Paenibacillus*	14.7% wl per 60d	-	-	-	Yuan et al., 2022a, 2022b
	*Alkali- resistant C. testosteroni*	-	More efficient degradation	-	-	Gong et al., 2018
Fungi	*Zalerium maritimum*	Reduction of mass and size, minimal nutrients	-	-	-	Paço et al., 2017
	*Aspergillus flavus*	Molecular weight degradation	-	-	-	Zhang et al., 2020
	*Phanerochaete chrysosporium*	-	-	-	9.42% wl per 1 year and 10d, ultraviolet (UV) pretreating	Jeyakumar et al., 2013
	*Engyodontium album*	-	-	-	18.8% wl per 1 year and 10d, UV pretreating	Jeyakumar et al., 2013
Animal	*Lumbricus terrestris*	Particle size significantly reduced, production of long-chain alkanes	-	-	-	Lwanga et al., 2017a, 2017b
	*Mealworm*	-	-	47.7% to CO_2_ conversion per 16d	-	Yang et al., 2015

Table 3 shows the importance of microorganisms in the degradation of MPs. It is important to note that biological methods can be coupled with other technologies, thus increasing the MPs removal. Adapting biological degradation to conventional systems poses challenges due to variations in the degradation efficiency of MPs, as a function of the strain type and its selectivity towards specific MPs. According to Table 3, the bacteria *Bacillus* and *Rhodococcus* exhibit high degradation percentages (wl%) for various plastics compared to other strains. For example, *Bacillus cereus* bacteria degraded PE in 40 days, while *Rhodococcus ruber* degraded PE in one week.

Also, from Table 3, it can be observed that fungus and animals also play a significant role in the degradation of MPs. For instance, the fungus *Zalerium maritimum* has been reported to reduce the mass and size of PE, while *Aspergillus flavus* can degrade the molecular weight of MPs. In the animal kingdom, *Lumbricus terrestris* (a type of earthworm) has been found to significantly reduce the particle size of PE and produce long-chain alkanes. Mealworms have also been reported to convert 47.7% of PS to CO_2_. These findings highlight the diverse range of biological organisms that can contribute to the degradation of MPs, each with their unique efficiencies. However, it is important to note that the degradation rates are generally low, indicating that MPs are resistant to biodegradation and that further research of these biological methods is required.

Moreover, various studies have highlighted the simultaneous use of multiple strains for MPs degradation and it has been stated that strain consortiums provide more effective MPs degradation than individual strains (Salinas et al., 2023; Shah et al., 2013). Seon et. al. (2019) carried out PE biodegradation by a microbial consortium, composed of a mix of *Bacillus sp*. and *Paenibacillus sp.* The authors observed that the microbial consortium accelerated the PE degradation compared to the biological efficiency of a single biotic strain. Huerta et al. (2016) reported the biological degradation of LDPE using mixed microbial consortiums, consisting of earthworm *Lumbricus terrestris* and bacteria isolated from the gut belonging to the phyla *Actinobacteria* and *Firmicutes*. The bacterial consortium from the worm gut effectively facilitated LDPE degradation. In light of these findings, some studies have begun to explore the use of transgenerational strains. These strains, passed down through generations, could potentially expedite recovery from chronic exposure to MPs and extend the survival of populations under threat (Martins & Guilhermino, 2018).

Fungus have been employed for the biological degradation of PE and PP (see Table 3). For example, Zhang et al. (2020) reported the utilization of specific isolated strains of *Aspergillus flavus*, derived from the gut contents of the wax moth *Galleria mellonella* for the biological degradation of PE. The authors declared the degradation of PE due to the weight loss of the MPs. Paco et al. (2017) use *Zalerium maritimum* for MPs degradation, and the authors observed a reduction of the mass and size of the MPs. Patrício Silva (2022) stated that *Phanerochaete chrysosporium* and *Engyodontium* albums were employed for the biological degradation of PP. In one year, and with a 10-day UV pretreatment, the former achieved a degradation rate of 9.42% wl., while the latter reached an 18.8% wl. degradation rate. Additionally, Yang et al. (2015) used the larvae of *Tenebrio molitor*, commonly known as mealworms, for the biodegradation of PS. The authors stated that 47.7% of the PS was converted to CO_2_ in 16 days, and the degradation was attributed to the formation of depolymerized metabolites in the larval gut.

Various studies have focused on enhancing degradation efficiency through biological methods with preconditioning treatments to promote biological degradation efficiency. For instance, Liu et al., (2023a, 2023b) reported that MPs previously exposed to photo-oxidation or UV-light exhibited superior biological degradation efficiency compared to non-irradiated MPs. The authors suggested that preconditioning treatments rendered MPs more sensitive or vulnerable to degradation by organisms. They observed the highest rates of oxidation and degradation of PP pretreated with UV-light for 10 days compared to non-pretreated PP. Khoironi et al. (2020) reported the biological degradation of PP pretreated thermally at 100 °C for 10 days. The authors observed a greater oxidation of the pretreated PP. It was established that photodegradation and subsequently chemical degradation promote the breakage of carbon chains, facilitating the biodegradation process. Scally et al. (2018) stated that conditioning MPs with non-thermal plasma (NTP) is effective. The authors found that pretreating LDPE with NTP discharges promotes biodegradation with *Pseudomonas aeruginosa*, attributed to chemical composition and surface polarity changes. In another sense, Jeyakumar et al. (2013) stated that the addition of starch to the reaction medium increases the MPs degradation by altering the pH to alkaline environments. The authors employed two different fungus, *Engydontium* album and *Phanerochaete chrysosporium*, to degrade PP in a reaction medium containing starch. The addition of starch showed a higher weight loss percentage than non-treated PP.

Additionally, the alteration of pH and the use of alkali-resistant strains have demonstrated enhanced degradation efficiencies, according to results of Auta et al. (2017). Gong et al. (2018) reported the modification of already-known strains through evolutionary engineering. The authors isolated *Commamonas testosteroni* and enhanced it to become an alkali-tolerant bacterium capable of thriving at pH = 12. They compared the biodegradation of PET treated with an alkali-resistant testosterone strain and *Comamonas testosteroni* under both high pH and neutral conditions. The results demonstrated that the PET decomposition strain under alkaline conditions not only grew faster but also exhibited a higher total biomass generated. Consequently, PET particles decreased more in size, and the MPs crystallinity changed to a greater extent.

Numerous studies have documented the isolation of biotic strains from several natural environments. For instance, Auta et al. (2018) successfully isolated strains from mangroves. Among the other eight isolated strains, *Bacillus cereus* and *Bacillus gottheilii* demonstrated significant %wl for PE, PET, and PS. In a separate study, Naudenforf (2016) isolated biotic strains from ocean waters, and conducted biodegradation experiments by immersing PP and PE in a bay for three months. The results were promising in the presence of *Pseudomonas*. However, it is important to note that not all biotas can adapt or recover from the presence of plastics. Supporting this, Martins et al. (2018) investigated the transgenerational effects and recovery in *Daphnia magna*, noting a decrease in the growth and reproduction rate of this specie in the presence of MPs.

The biodegradation of MPs using bacteria, fungi, and invertebrate annelids promotes significant degradation efficiency, though this process generally is a slow process. Plastic-degrading bacterial genera include *Bacillus*, *Pseudomonas*, and *Rhodococcus*, have shown potential in the degradation of PE, PET, and PS. Some strains, like the *Bacillus* and *Rhodococcus* species, could even degrade PE and PP. However, their biodegradation rates are mostly low and vary within the range of 0–15%. Some fungi species such as *Zalerium maritimum* and *Aspergillus flavus*, in addition to species like invertebrate annelids such as earthworms (*Lumbricus terrestris*) and mealworms, could contribute to the MPs degradation through size and mass reduction towards conversion into CO_2_, these processes are equally slow. Synergistic approaches, like microbial consortia combining different strains such as *Bacillus* and *Paenibacillus* or microbial consortia from earthworm guts, have shown an effective accelerated MPs degradation for PE and LDPE.

Pre-treatment methods such as thermal heating, UV irradiation, or non-thermal plasma might improve the efficiency of microbial degradation by making the MPs structure more susceptible to breakdown. For example, it was discussed that UV light-treated PP and preheated PP were degraded to a greater extent than in conventional biodegradation processes. In the same sense, PET degradation was enhanced by using an alkali-resistant strain, *Comamonas testosteroni*, due to alkaline conditions promotes microbial growth. Also, isolation of bacterial strains from different natural environments, like mangroves and oceans, has resulted in the discovery of microorganisms that can degrade various types of MPs. Although adaptation to plastic pollution for some species is still a challenge, the deep study of such strains holds promise for improving MPs biodegradation strategies.

In summary, bioremediation focuses on using biological methods to help disintegrate MPs and eliminate them in the water samples. Microbial decomposition is based on the natural capability of bacteria, fungi, algae, and even worms to degrade MPs particles. Such microorganisms can feed on MPs and generate biomass as resulting products in a continuous cycle. Despite the promises of biological approaches, challenges still persist in view of the slow degradation rates and the complexity of improving such processes for large-scale applications. However, biological treatments offer an environmentally friendly alternative for MPs removal, especially when combined with other treatment technologies.

### Chemical methods

**Fig. 10:**
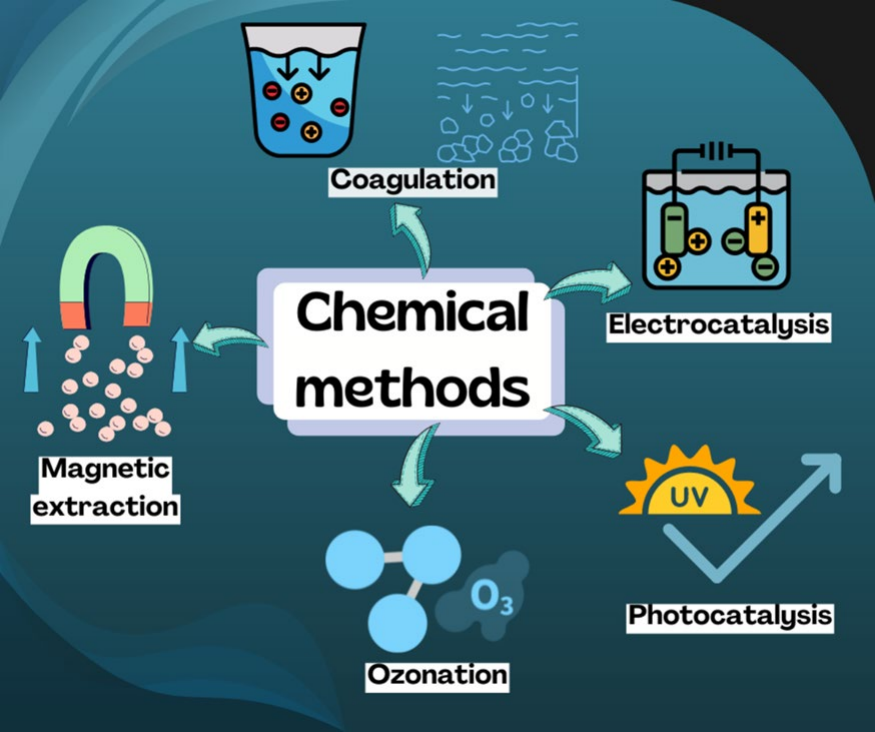
Chemical methods for MPs degradation or removal

From recent decades, MPs removal from water has become a priority and the use of chemical methods has shown to be an interesting alternative. Chemical processes are widely used in the industry for the removal of compounds in water and therefore some similar principles are used to remove or degrade MPs through several techniques, which mainly include changing the pH of the reaction solution, changing the polarity of the MPs surfaces, coagulation, electrocoagulation, photocatalysis, magnetic extraction using Fe nanoparticles and ozonation. In the following sections, several chemical methods for the treatment of water containing MPs will be presented. The removal rates and critical operating conditions of these techniques will be discussed. Figure 10 shows the chemical methods commented in this section: coagulation, electrocoagulation, photocatalysis, magnetic extraction and ozonation.

Chemical methods for removing MPs from water have shown significant potential but also present several challenges. These techniques, such as coagulation, electrocoagulation, photocatalysis and ozonation are based on chemical reactions or changes on the chemical environment to either degrade or remove MPs from water. However, their effectiveness can be influenced by factors like the type of chemical agents used, the size of the MPs, the pH of the aqueous environment or the concentration of the MPs and other pollutants. While chemical methods can provide high removal efficiencies, they often suffer from limitations such as high energy consumption, the generation of secondary pollutants, and the need for specialized equipment. A critical gap in these methods is their environmental sustainability, which needs to be addressed to ensure that these technologies can be widely adopted for large-scale water treatment.

#### Coagulation

**Fig. 11:**
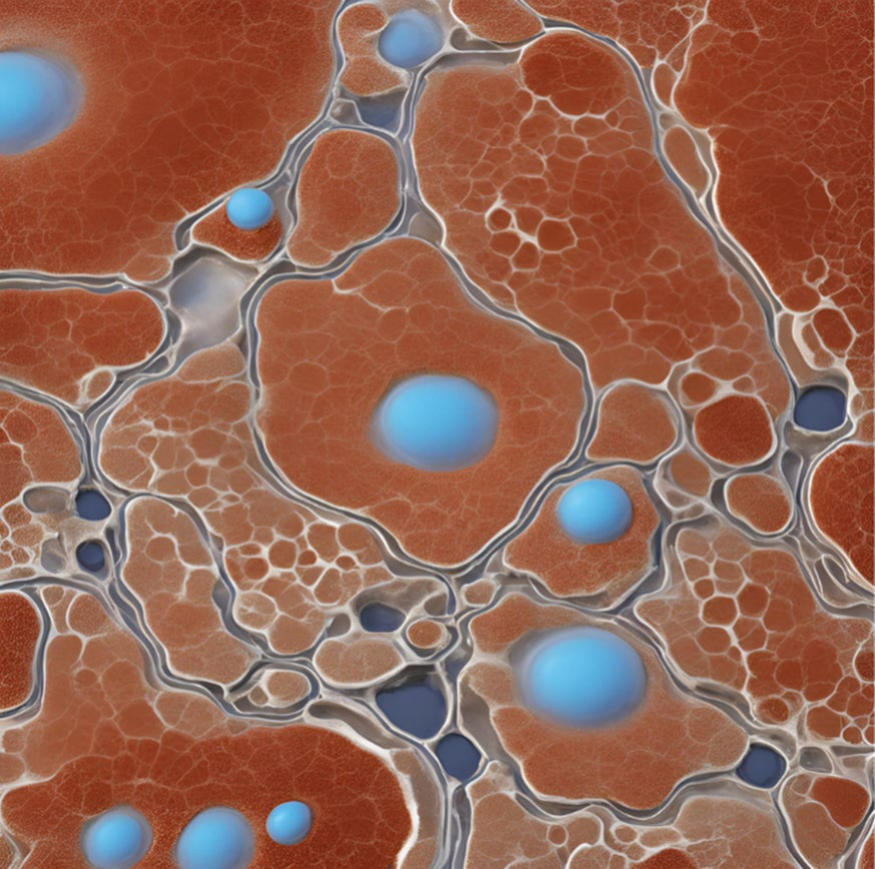
Graphical representation of the coagulation process of MPs

Coagulation is the process where chemical agents are introduced into the aqueous environment to affect the polarity of the MPs particles, which causes them to agglomerate together and, therefore, lead to sedimentation, enhancing the removal of the MPs particles from the water. Figure 11 shows a graphical representation of the coagulation process, that is, the illustration seeks to represent the links between the MP particles, due to the coagulating compounds, which will allow the formation of a coagulum that will tend to sediment and therefore promote the separation of MPs from the aqueous medium. Thus, the coagulation method uses several coagulants to alter the physical surface properties of dissolved and suspended solids to facilitate their removal by sedimentation. MPs can be effectively removed through the coagulation/sedimentation process. However, the MP removal efficiency is a function of the agent coagulant used, which can be explained by classical coagulation removal mechanisms, such as charge neutralization, adsorption, and sweep flocculation (Shammas et al., 2021). On the other hand, the addition of extra chemicals in water and wastewater can cause more unwanted side products, which become more difficult to treat by traditional methods (Monira et al., 2021).

In this sense, Sharma et al. (2021) used Al- and Fe-based salts to promote the coagulation of MPs. The Al-based salts showed a better performance compared to Fe-based salts. After salt addition, the MPs were agglomerated by sedimentation. The study showed that the MP removal efficiency was approximately 40% when the MPs size was lower than 0.5 mm and using 20 mg/L of AlCl_3_. Rajala et al. (2020) reported the use of ferric chloride (FeCl_3_), poly aluminum chloride (PAC), and polyamine as coagulants salts for MP removal. The highest removal efficiency (99.4%) was obtained by using FeCl_3_ and PAC, while polyamine presented the lowest efficiency. According to the authors, the best removal efficiencies were obtained in the pH range of 6.8—7.5. Also, a relationship between the MPs removal efficiency and the size of the MPs particles was reported, as a 95% and 76% removal efficiency were observed for particles at 1 mm and 6.3 mm, respectively. Khan et al. (2023) reported the use of Al- and Fe-based salts for the removal of PE and polyacrylamide (PAM). It was observed better performance using Al^3+^ ions. Also, it was stated that PAM was removed more efficiently than PE. The removal efficiency increased with smaller particles (less than 0.5 mm) up to 61.19% at a pH of 7.

Skaf et al. (2020) performed conventional coagulation tests for the removal of MPs with shape of spheres and fibers. They used aluminum sulfate 18-hydrate (Al_2_(SO_4_)_3_−18H_2_O), commonly known as a coagulant alum. Pure kaolin was used to simulate the presence of inorganic colloidal clays typically found in drinking water. Jar-tests were conducted using simulated drinking water and tap water containing kaolin or MPs. These studies confirmed that the conditions suitable for the removal of kaolin are also effective for the removal of the spheres of MPs. The results indicate that conventional coagulation is suitable for the removal of certain MPs from solutions. Therefore, these statements are basic to the electrocoagulation setup and the choice of optimal electrodes as is stated by Liu et al., (2023a, 2023b).

Coagulation is a widely used chemical method for MPs removal, which is based on the addition of coagulants to the water or wastewater volumes, promoting changes in the chemical properties of suspended particles of MPs. The main effect of the coagulants is the agglomeration of MPs and later they are removed by sedimentation. While effective in the treatment of smaller MPs, the removal efficiency significantly decreases for larger particles, which creates a challenge for treating diverse water samples. Another important aspect is the nature of the coagulant used, i.e., Al-based salts provide better performance compared to Fe-based salts. Coagulant addition could present a possible environmental complication because of the toxic by-products may complicate the further water treatment. A lot of studies need to be done to find coagulants that are effective across a wide range of MPs (size and form) and several water condition (competitive pollutants and several pH values). Also, further studies should be developed to implement methods to mitigate unwanted side products.

#### Electrocoagulation

**Fig. 12:**
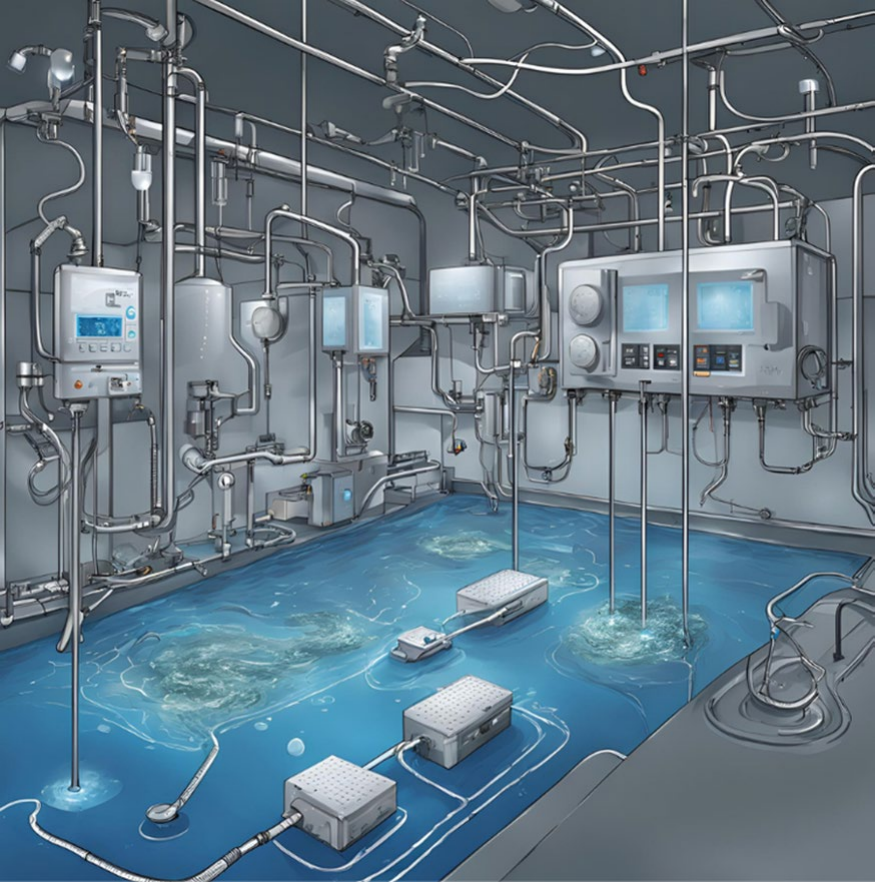
Conceptual implementation of the electrocoagulation process for MPs removal

Electrocoagulation is a coagulation process assisted by an electrical field under the presence of metal electrodes, which are made of cost-effective and non-toxic materials such as aluminum, iron, and stainless steel (Liu et al., 2010). That is, an electrolytic reaction on the surface of the working electrode allows the generation of ions into the reaction. The generated Fe^2+^ or Al^3+^ ions promote the formation of microclots with the suspended MPs particles to create flocs. The electrocoagulation process, in contrast to conventional coagulation, does not need the addition of chemicals at the reaction system, since the microcoagulant particles are released by the electrocatalytic reaction on the surface of the working electrode. However, the amount of electricity required is high, and the electrodes must be periodically replaced (Barrera-Díaz et al., 2018). Figure 12 shows a schematic illustration for the conceptual implementation of the electrocoagulation process for MPs removal. Since the process requires only a working and reference electrode, it can be considered a large space to treat a very large volume of water by the electrocoagulation process.

Xu et al. (2021) removed PE of several particle sizes in a pH range of 3—10, where the optimal removal value was founded at pH = 7.5 with 99.24% for MPs removal, using FeCl_2_ and AlCl_3_ as coagulant salts and metal electrodes. The authors observed that the removal efficiency increased from 11.56% to 17.23% at pH 6 and 8, respectively, when the size of the MP particles was less than 0.5 mm. Elkhatib et al. (2021) achieved a removal of up to 98.5% of MPs when the current density of the electrocatalytic system was 8.07 mA/cm^2^, using aluminum electrodes with a pH range from 2 to 7. However, despite the efficiency of the electrocoagulation process, it is important to note the negative impact from the costs associated with the equipment and its operation as is mentioned by Liu et al., (2023a, 2023b).

Kasmuri et al. (2024) used wastewater obtained from the Universiti Teknologi MARA and a rectangular reactor with a working volume of 15 L, with aluminum electrodes. They removed PE using electrocoagulation, obtaining an efficiency up to 82%. Subair et al. (2024) made a study systematically examining the impact of different combinations of aluminum (Al) and stainless steel (SS) electrodes, including Al-Al, SS-SS, Al-SS, and SS-Al. Among these combinations, it was found that the Al-Al pairing exhibited outstanding efficiency for MPs removal, while simultaneously minimizing energy consumption. Initial pH emerged as a critical parameter, with a neutral pH of 7 demonstrating the highest removal efficiency. This refined system exhibited remarkable proficiency in eliminating MPs of several size ranges (0–75 μm, 75–150 μm, and 150–300 μm), achieving removal efficiencies over 90%. Sezer et al. (2024) observed that the 500 mL of samples taken from the food packaging industry contained an average of 43 ± 7 MPs of different sizes and as fiber structures. It was observed an optimal degradation in 12 min by electrocoagulation process. According to the results, the highest MPs removal efficiency was achieved at pH of 6.74, with 99% of MPs removal.

Electrocoagulation uses an electrical field to generate coagulant salts (ions) on the surface of the metal electrodes for efficient removal of MPs from water. Several advantages of this technique over the traditional coagulation process approach include avoiding addition of chemical additives and treating diverse water samples. The efficiency of the electrocoagulation process is a function of several factors such as electrode material, current density and pH, leaving a gap in standardizing optimal operating conditions. However, there are significant drawbacks to electrocoagulation, including high energy consumption and frequent electrode replacement, making the process costly and not very suitable for large-scale applications. Improvement in the process will require research into more energy-efficient systems and more durable electrode materials.

#### Photocatalysis

**Fig. 13:**
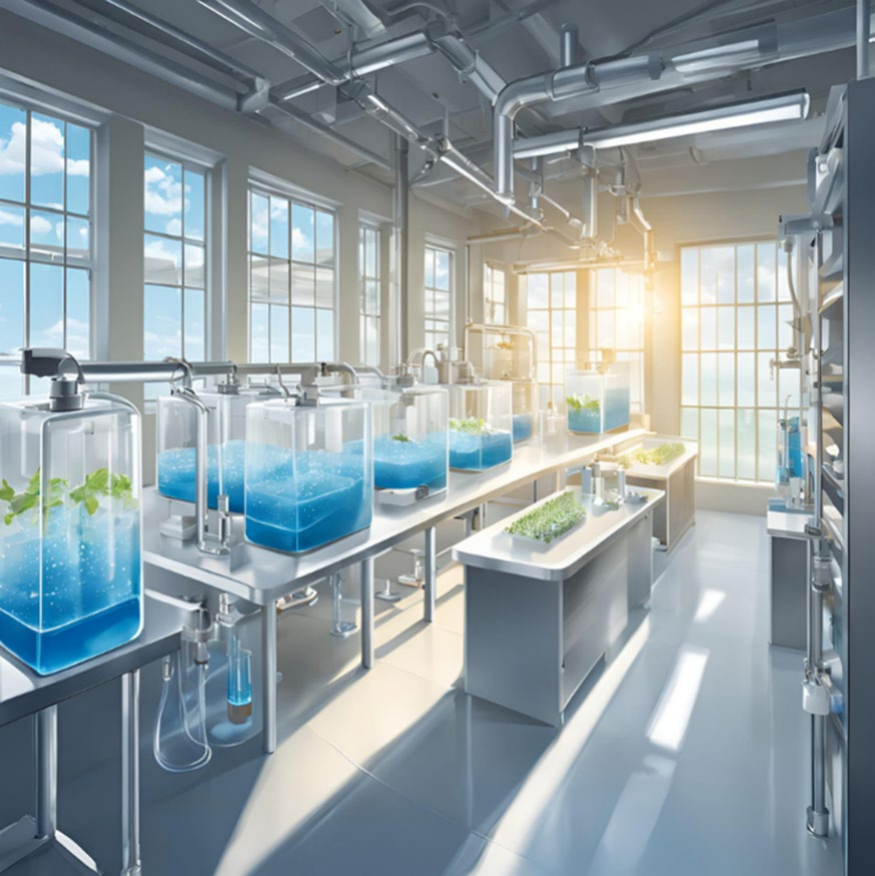
Conceptual design of photocatalytic laboratory set-up

Photocatalysis is a technology that involves a source of light or radiation and a photocatalyst that accelerates a photochemical reaction. These reactions can be either the reduction of inorganic ions or the oxidation of organic compounds in water bodies, such as MPs (Li et al., 2020). In heterogeneous photocatalysis, •OH^−^ and •O_2_^−^ radicals are generated at the surface of the photocatalyst; therefore, the oxidation reactions are due to the attack of radicals towards organic/inorganic matter and MPs (Lee et al., 2016). Figure 13 shows a conceptual representation of a design of photocatalytic laboratory set-up. The MPs degradation by photocatalysis is a semicontinuous or batch process due to the nature of the light used. Ultraviolet (UV) irradiation or visible (vis) irradiation can be used as a function of the nature of the photocatalyst. That is, some semiconductor materials or composites are photoactive under UV- o vis-irradiation. Therefore, irradiation sources will determine the water or wastewater volume to be treated.

Some researchers have developed novel materials for photocatalysis processes. For example, Wang et al. (2019) used a TiO_2_ catalyst under UV light for MPs removal, achieving 67% for MPs degradation. Tseng et al. (2010) and Ge et al. (2022) used a TiO_2_ catalyst, synthesized by sol–gel with titanium isopropoxide as a precursor, to degrade MPs. The maximum MPs removal, at pH = 3 and 0 °C, was 99%. Du et al. (2021) compared different catalysts, such as polyacrylamide-grafted ZnO, ZnO-Pt, TiO_2_, NiAl_2_O_4_, TiO_2_-NPs, TiO_2_-NTs, C coated TiO_2_, Mo-TiO_2_, C, N, TiO_2_, N-TiO_2_, etc. Where the operating conditions for the MPs degradation such as light source, degradation time, and water source were modified to determine the removal of PP, PE, PS, LDPE, and HDPE. The biggest MPs degradation at 12 h under UV light was obtained with the TiO_2_-NP, with a degradation up to 98%. For HDPE degradation the C, N-TiO_2_ catalyst was the most efficient method with degradation up to 70% with similar operating conditions under visible light during 50 h. For PE degradation the TiO_2_-NTs was the most effective catalyst, with an efficiency of 67% under both UV and visible light during 15 and 45 days, respectively. For PP, ZnO was the most efficient catalyst, with degradation up to 65% using UV light for over 500 h.

Xu et al. (2021) compared different catalysts such as TiO_2_, sol–gel N-TiO_2_, C, N-TiO_2_, ZnO, and protein-derived NTiO_2_, where the operating conditions for the MPs degradation such as light source, degradation time, and water source were modified to determine the removal of PS, PP, and PE. Du et al. (2021) obtained the biggest removal for PE using visible light with a degradation time of 50 h, pH = 3, and T = 0 °C, with a degradation efficiency over 70%. Using ZnO catalyst they obtained degradation efficiencies of 65% under visible light with a degradation time of 456 h. Using TiO_2_ catalyst the authors could degrade PE with a efficiency of 44% using UV light with a degradation time of 12 h.

Other authors, such as Prasad et al. (2020), studied CuO nanocrystals as photocatalysts, obtaining the degradation of MPs such as PE, obtaining removals of up to 97% in a 2 h under visible light at T = 23 °C. Zhong et al. (2024) used FeCl_3_ as a photocatalyst and polyamide 6 fiber as simulated MPs to study the photodegradation performance of polyamide-MPs in model water. The results showed that polyamide PA6 fiber could be degraded in a 50 mmol/L FeCl_3_ solution under sunlight, and the degradation rate could be close to 100% after 10 days of irradiation. Through experimental comparison, it was also found that Fe^3+^ played a major role in the system, and other metal salts had no degradation effect.

Llorente et al. (2020) studied the effect of the size particle in the removal efficiency, where it was found that photocatalysis of MPs (LDPE and HDPE) with a potential application in WWTPs is possible, although some operational parameters such as MPs particle size and shape should be considered for the design of effective and fast processes. The study used an N-TiO_2_ catalyst under visible light at pH = 3 in time lapses of 50 h, obtaining the best degradation with a size particle of 382 ± 154 µm and spherical shapes.

Photocatalysis has proved to be one of the most promising chemical ways to remove MPs, where it is used a solid photocatalyst and light to generate radicals that oxidize MPs particles, promoting their decomposition, in addition to the degradation of organic or inorganic compounds contained in the water volumes. TiO_2_-based materials are commonly used as photocatalysts since they have shown to be effective to produce active radicals for the degradation of MPs. Some limitations still exist to the development of more efficient photocatalysis processes such as the design of more photoactive catalysts, the conceptualization of processes to be able to specify the particle size of MPs to be treated and the conditions of exposure to light as well as its sources. Besides photocatalytic degradation of MPs has shown promising results, the scalability of this technology regarding its economic viability is still a challenge to overcome. Therefore, further research must be carried out for the development of faster and more efficient catalytic systems that work under real conditions, maintaining sustainability and low costs.

#### Magnetic extraction

**Fig. 14:**
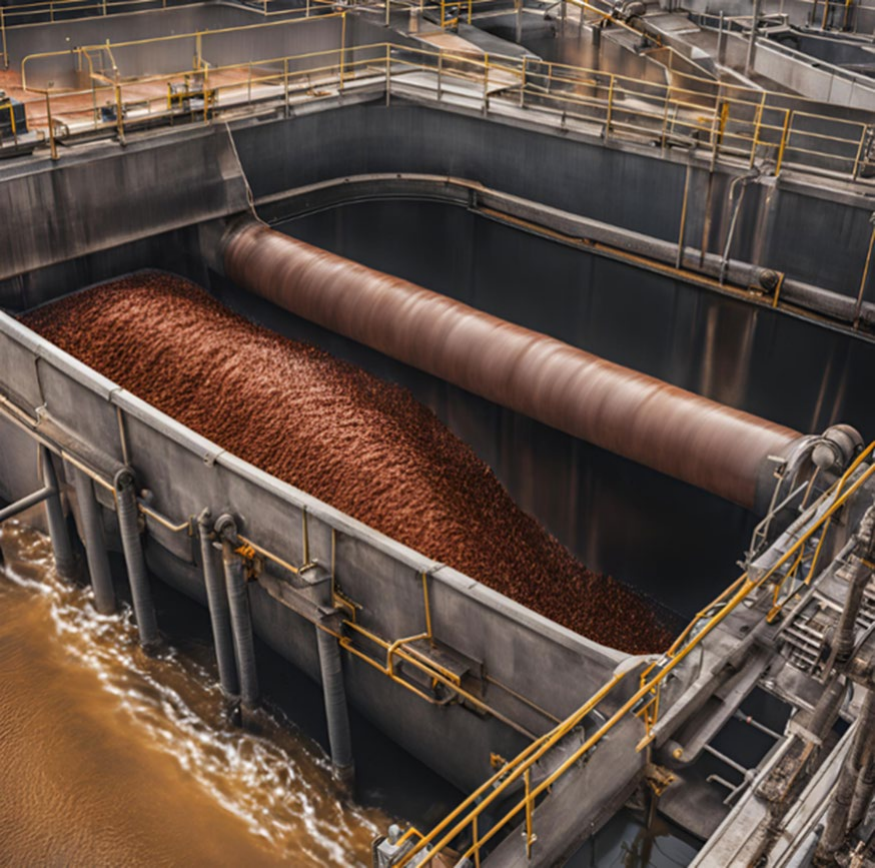
Schematical representation of MPs removal by magnetic extraction

The operational principle of magnetic extraction resides in the magnetization of the hydrophobic surface of MPs by binding with Fe nanoparticles. Subsequently, MPs with a functionalized surface can be separated from the water bodies by an external magnetic field (He et al., 2021). Figure 14 shows a schematical representation of MPs removal by magnetic extraction. It can be observed the agglomeration of MPs particles which are separated by an external magnetic field outside the water volume.

Among several case studies, Grbic et al. (2019) reported the removal of small (< 20 μm), medium (20 μm-1 mm), and large (1 mm to 8 mm) MPs from fresh seawater by magnetic extraction using a N52-grade neodymium magnet. The magnet was attached to a stainless-steel rod, dipped and swirled in the sample and, after the magnet was removed and rinsed with deionized water, the process was repeated. The authors used a solution of hexadecyltrimethoxysilane-methanol with Fe nanoparticles (25 nm). The samples were taken from seawater in California. The MPs recovery changed depending on the particle size. For large particles, recovery was 96% for PP, 92% for PVC, 97% for PU, 96% for PS and HDPE, and 74% for PET. For medium size particles, removal was 89% for PP, 59% for PVC, 73% for PU, 100% for PS, and 100% for PE. In the case of small particles, the removal was 88% for PS and 96% for PE. Hamzah et al. (2021) added oil into a MPs suspension (model solution) with magnetite powder (Fe_3_O_4_, 0.50 g/L) to form a ferrofluid. Several oil volumes (0.5, 1.0, 1.5, 2.0, and 2.5 ml/L) and Fe_3_O_4_ dosages were assessed. The results show excellent organic load removal, but merely 64% for MPs removal. In a similar study, Zandieh et al. (2022) added 1% (wt./wt.) of Fe_3_O_4_ powder with respect to the total amount of MPs. The MPs recoveries were close to 99%. Pasanen et al. (2023) also used Fe_3_O_4_ powder to remove the MPs from a wastewater sample with 25 mg/L of MPs. The MPs removal by magnetization in this process reached 94% efficiency, which is an acceptable value but lower than other similar studies which report the use of Fe_3_O_4_.

The Magnetic extraction method allows the removal of MPs through the binding of MPs to Fe nanoparticles, and later the complex gets magnetized and separates by means of an external magnetic field. It has been reported that large-sized MPs such as PP, PVC, PU, PS, and HDPE can be efficiently removed from water volumes with recovery rates as high as 96%. The effectiveness of magnetic extraction is significantly influenced by the size and surface area of the MPs, that is, smaller MPs bind to the magnetic nanoparticles much more effectively than greater MPs, hence giving way to a higher recovery rate. However, challenges still remain in scaling this process up for larger particle sizes and addressing the need for significant amounts of Fe nanoparticles for effective recovery. Nevertheless, magnetic extraction is a promising methodology because of its high effectiveness and negligible amount of residue produced. It could be a potential option for the mitigation of the contamination of microplastic in water.

#### Ozonation

**Fig. 15:**
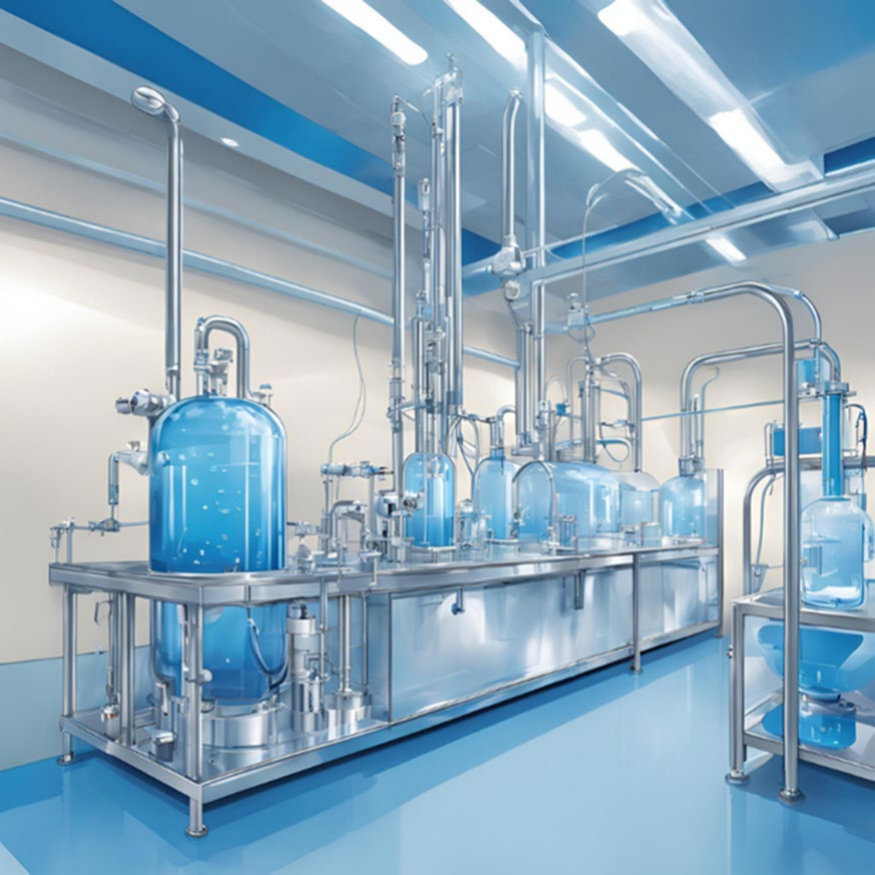
Representative scheme for the MPs degradation by ozonation process

Ozone is produced when oxygen molecules (O_2_) are dissociated by an energy source, producing oxygen atoms that subsequently collide with an oxygen molecule to form an unstable gas, ozone (O_3_). Since ozone is about ten times more soluble in water than oxygen, high concentrations of ozone may be obtained by saturating water with an ozone/oxygen mixture from an ozone generator. In micropollutant abatement, for example, the reactivity of a micropollutant determines the efficiency of its elimination by ozone treatment (Wei et al., 2017). Ozone is a highly reactive molecule that can attack the chemical structure of the MPs, degrading them and significantly reducing their lifespan. The MPs degradation is proportional to the amount of ozone, but even low ozone concentrations at room temperature can cause significant degradation over time. Ozonation is usually used as pretreatment to change the chemical structure of the MPs to make them more susceptible to biodegradation; however, wastewaters with high concentrations of organic pollutants could generate toxic byproducts from the pollutant partial degradation (Jianlong & Chen, 2020). Figure 15 shows a representative scheme for the degradation of MPs by ozonation process. In this proposal, it can be observed that ozone gas, from an ozone generator, can be bubbled in the volume of water or wastewater to reach the saturation of the liquid. The ozone reacts with MPs, degrading them, in a semicontinuous or batch process.

In this sense, Fitri et al. (2021) treated PE particles with ozone (5 L/min) at pH = 12 in 3-h cycles. The surface analysis of PE particles before and after the ozone treatment indicated that the structure of the MPs changed due to the disappearance of some functional groups and the apparition of carbonyl groups (aldehyde, ketone, alcohol, and carboxylic acids) after the ozonation process. Yang et al., (2021a, 2021b) used samples from landfill leachate in Belgium, placing them in a 3L batch reactor at pH = 8.5 and 20 °C. The O_3_ flowed through the reactor at 0.95 gO_3_/gDOC (Dissolved Organic Carbon). The operating cycles were 90 min long and achieved low MPs removal. Hidayaturrahman (2019) used a dosage of 12.6 mgO_3_/L to treat wastewater samples obtained from a domestic WWTP in South Korea. Also, the sample was irradiated for 1 min. Ozone technology significantly reduced the number of MPs, as > 91% of MPs were removed from their samples. Although chemical methods have advantages in terms of removal percentages due to their reaction kinetics and conversion into other substances, other factors should not be forgotten, such as operating costs, which can be an impediment when implementing new water treatment technologies. Therefore, in order to choose the appropriate treatment technology, it is necessary to know the nature of the influent to be treated in order to make the best decision in terms of cost–benefit ratio and thus release the least number of pollutants possible, in compliance with the national standards of each country.

Ozonation is a chemical method to degrade MPs in water or wastewater volumes that uses ozone (O_3_), a highly reactive molecule, generated by the dissociation of molecular oxygen. Ozone molecules attack MPs particles and break down their chemical structure. This technique is effective for MPs degradation even at low concentrations of ozone, however research in the field indicates that degradation is proportional to the amount of ozone used. This method has been widely used as a pretreatment step to degrade the chemical structure of the MPs, since it has been stated that biological degradation allows greater degradation of the MPs after ozonation processes, that is, the MPs become more biodegradable. However, toxic byproducts may be produced during ozonation, especially in high organic-loaded wastewater, creating a potential risk to the environment. In addition, although ozonation has been successful in changing the structure of MPs, complete degradation is not achieved. It has been indicated in various studies that ozonation can effectively reduce microplastics concentration in water, however since this process promotes the formation of harmful byproducts, the large-scale water treatment remains a challenge to overcome.

Table 4 shows a comparison of chemical water treatment methods, and their MPs removal efficiency reported. The data compares different methods, taking as a reference the pH of the aquatic environment, as well as the ideal concentrations for maximum MPs removal, in addition to listing limitations and benefits.

**Table 4:** Comparison of chemical methods for MPs removal

Method	Description	pH	Concentration	Contaminant removal (%)	Limitations	Benefits	Reference
Coagulation	Removal of PE MPs using iron and aluminum salt coagulants and ultrafiltration. The experiments were carried out under different concentrations of Al^3+^ and Fe^3+^ ions, and the results indicated that Al^3+^ has better performance than Fe^3+^. PAM increases efficiency	7	Low concentration of Al coagulant, 0.5 mM	25.83% without PAM and 61.19% with PAM	Addition of extra chemicals	Efficient at removing small particles, manageable operation control	Padervand et al., 2020
	Ferric chloride, PAC, and polyamine were used to remove MPs. The study shows that the highest removal efficiency obtained was 99.4%, and ferric chloride and PAC were more efficient than polyamine. Ferric chloride and PAC displayed similar efficiencies. There is a strong dependence on pH	6.8–7.5	The required dosage for 90% MP removal at pH 7.3 was 0.37 mmol/L for iron and 0.16 mmol/L for aluminum	High MP removal, above 95% was observed for 1 µm MPs, and above 76% for 6.3 µm MPs	Larger sizes of MPs showed lower efficiencies	-	Pramanik et al., 2021
	Al- and Fe-based salts were used, and it was observed that coagulation could eliminate MPs that are expected to float. The Al-based salts also showed better performance in comparison to Fe-based salts. The smaller the PE particle size, the higher the removal efficiency, with MPs smaller than 0.5 mm exhibiting maximum removal efficiency	5.5—7	For low coagulant dosage (AlCl_3_·6H_2_O 0.5 mM)	Maximum removal 40%	Fouling gradually formed when the dosage of Al and Fe salts was increased	-	Ma et al., 2018
	The occurrence of MPs in different drinking waters (before and after treatment) from three different water treatment plants in the Czech Republic were studied	7.5	AlCl_3_⋅6H_2_O, 30 mg/L	The removal rates of MPs in WWTP1, WWTP2, and WWTP3 were 70%, 81%, and 83%, respectively	-	-	Xu et al., 2021
Electro coagulation	The process of the formation of flocs from cations generated by metal electrodes under the action of an electric field, which mainly includes the formation of “micro-coagulants”, losing stability of suspended particles under the action of coagulants, and the formation of flocs through the interaction of coagulants and particles. Removal of PE MPs from wastewater using EC in a stirred-tank batch reactor	7.5	The optimum NaCl concentration used is 0–2 g/L	Efficiency was above 90% over a wide pH range (pH = 3–10). The removal efficiencies under acidic (pH 3) and alkaline (pH 10) conditions were lower than under neutral conditions, and the optimum value of 99.24% was obtained at pH 7.5	Still requires more pilot scale conditions to test instead of lab conditions	Coagulation happens in situ, in contrast to chemical coagulation, which makes it easier, there is no chance of secondary pollution and much less sludge creation, therefore, eco-friendlier	Xu et al., 2021
	Electrocoagulation consists of three significant events: electrolytic reactions on the electrode surface, coagulant formation in the medium and adsorption of colloidal/soluble contaminants through a coagulant, and finally, separation by sedimentation due to the formation of hydrogen bubbles by cathodes that assist particle separation. The study was carried out on microbeads, not specifically MPs	7.5	NaCl concentrations (2–8 g/L) were studied but there was no significant change	A removal efficacy of > 89% was attained at pH values going from 3 to 10; nevertheless, the efficacy at pH 3 and 10 was lesser as compared to pH 5, as well as pH 7.5. A maximum of 99% removal was observed at pH 7.5	Some limitations in the technology, such as the replacement of the anodes and electricity requirements	Eco-friendly water treatment technology	Sharma et al., 2021
Photocatalysis	Oxidation process that can degrade pollutants into water using highly oxidizing species (⋅OH^−^, ⋅O_2_^−^) generated by the photocatalysts. The method uses the photocatalytic degradation of HDPE MPs using two photocatalysts based on N- TiO_2_ (i.e., protein-derived N-TiO_2_ and sol–gel N-TiO_2_). The protein-derived showed better results, as well as being exposed to simulated UV radiation	3	-	The degradation efficiency of 5 mm PS MPs in the liquid phase reached 44.66% after 12 h of illumination under 254 nm UV, which further increased to 99.99% in the solid phase under the same conditions because the adverse effect of the hydrophilicity of PS MP in the aqueous solution in the reaction was avoided	Most of the current studies on the photocatalytic degradation of MPs are conducted in single photocatalytic systems, which could be limited due to the rapid recombination of the electron (e^−^) hole (h^+^), improper band values and positions, and slow surface reaction kinetics, resulting in low performances. No cost-effective studies. At present it is not possible to achieve full degradation, therefore producing several intermediates which may be toxic	High efficiency in experiments	Xu et al., 2021
Magnetic extraction	Coated Fe-nanoparticles used to magnetize plastics, allowing magnetic extraction and the isolation of MPs. A key characteristic of this method is that its effectiveness is relative to the surface area to volume ratio	-	For large MP tests, a suspension containing ~ 2 mg of modified Fe nanoparticles was added to ~ 200 mL of artificial seawater	The average recoveries were 96% ± 6% for PP, 92% ± 7% for PVC, 105% ± 8% for PU, 96% ± 7% for PS, 96% ± 7% for HDPE, and 74% ± 9% for PET	Bigger particles may not show the same efficiency	For smaller particles, more of the Fe nanoparticles can bind per unit mass of plastic. Thus, this method is particularly useful for small MPs (< 20 53 µm)	Grbic et al., 2019
	Magnetic fields can be employed to separate MPs grafting with magnetic seed particles. This approach has the advantages of an enormous capacity, less waste sludge, and separation enhancement owing to long- range magnetic force. Magnetic separation has been applied to remove nanomaterials from wastewater. Magnetic extraction consists of three stages: preparing magnetic seeds, enhancing magnetic seeding-induced aggregation, and improving separation efficiency	-	-	The researchers extracted 92% of 10–20 µm MPs (PE and PS beads), 93% of > 1 mm MPs (PE, PET, PS, PUR, PVC, and PP), 81% of 0.2–1 mm MPs (PE, PS, PUR, PVC, and PP), and 78% of 0.2–1 mm MPs (PE, PS, PUR, PVC, and PP) from seawater, freshwater, and sediments, respectively	Large amount of magnetic seeds required, separation of magnetic seeds and particles	Less waste sludge, high efficiency, high volume	Yang et al., 2021a, 2021b
Ozonation	Changes in the chemical structure of PE MPs along with the influence of pH, ozone flow rate, and duration of contact between ozone and MPs	12	-	The optimum operating condition appeared at pH 12 with 5 L/min ozone flowrate, resulting in 0.0482% weight loss and carbonyl bond intensity reached 104.556% after 3 h of ozonation	As it mainly changes the chemical structure, this process is mostly used as a pretreatment	-	Fitri et al., 2021

## Conclusions and outlook

This manuscript sets the scene of the global environmental problem of the microplastics (MPs) occurrence and their distribution throughout different aquatic environments. MPs, considered as emergent pollutants, should be removed or degraded from the aquatic environments due to the toxic effects on aquatic animals and humans. The removal of MPs from water and wastewaters environments arises as an imperative measure to control the excessive increase of these. On the one hand, a lot of organizations, companies and several social sectors do not follow government policy for the management and disposal of MPs. On the other hand, MPs are generated naturally due to the extensive quantity that already exists in all ecosystems. Potential emergent technologies for the MPs removal mainly are physical, biological, and chemical methods. The presented perspective on the current innovative technologies was commented on emphasizing the existence of emerging methods rather than an abundant description of the fundamentals and bases of these methods. Potential physical methods for MPs removal are as adsorption, filtration and flotation. Adsorption is a separation technique to remove MPs from water using adsorbent materials, that is, the MPs particles are deposited (physical or chemical processes) on the surface of adsorbent materials. Currently, the most common adsorbents are based in oxides metallics, carbon-based materials or biomass-based materials or the combination of all the above. Two aspects are the limiting steps: 1) the limited capacity of the adsorbent materials and 2) the size or shape of the MPs particles. Without a doubt, researchers are seeking to develop novel and next-generation materials that could overcome the aforementioned limitations. The ultrafiltration and dynamic membranes can be an excellent alternative for the removal of MPs since the above two methods allow the selective retention of MPs while the water to pass through freely. The highly efficient separation is based on their size of the MPs particles. The main problems to overcome for these technologies are the operational costs, membrane fouling and the minor effectiveness for small-sized particles. In addition, further studies to enhance their scale-up and efficiency to bigger water volumes. Flotation techniques such as mechanical flotation, dissolved air-assisted flotation and electro-flotation allow the MPs removal by the attach of suspended MPs particles to ascendent air bubbles to make the particles float. The main characteristics to improve flotation methods are the bubble size and the MPs particle density and shape. An interesting combination of flotation methods with membrane technologies promises potential MPs degradation. However, further research for the improvement of bubble characteristics is needed to the large-scale water and wastewater treatment.

Biological methods using microalgae strains such as Chlorella pyrenoidosa, Cylindrotheca closterium, and Dunaliella salina have been used satisfactorily for the degradation of MPs. In addition, a combination of microalgae with sunlight, CO2, and nutrients allow polish even more the water and wastewaters volumes. Although degradation is hampered when MPs exceed certain sizes. Bacteria such as Bacillus, Pseudomonas, and Rhodococcus have been used to the degradation of PE, PET, PP and PS obtaining a wight loss in the range of 0–15%. Fungi species such as Zalerium maritimum and Aspergillus flavus or earthworms (Lumbricus terrestris) and mealworms have also degraded MPs particles achieved size and mass reduction and conversion into CO2. Also, microbial consortia from earthworm guts or Bacillus and Paenibacillus have shown an effective PE and LDPE degradation. A combination of biological methods with thermal heating, UV irradiation, or non-thermal plasma improved the efficiency of microbial degradation by making the MPs structure more susceptible to breakdown. In other perspective, alkali-resistant strains or the isolation of bacterial strains from mangroves and oceans have also been reported as accelerators of the degradation of various types of MPs. Although biological degradation approaches are promising, large-scale applications must overcome slow degradation rates.

Chemical methods for MPs removal such as coagulation are based on the addition of Al-based salt or Fe-based salts coagulants to the water or wastewater volumes promoting the agglomeration of MPs and later, they are removed by sedimentation. The removal efficiency significantly decreases for larger particles. Coagulant addition could generate toxic by-products that may complicate further water treatment. In the electrocoagulation method, the coagulant Al- or Fe-ions are generated at the surface of a metal electrode. MPs removal from water samples is successfully reached. The electrocoagulation process allows the treatment of water or wastewater volumes in a wide pH range and the addition of chemical additives is avoided. The main drawbacks are the high energy consumption and frequent electrode replacement, generating high costs for large-scale applications. Photocatalysis is a very promising chemical method for degradation of MPs. It is necessary to have a photoactive solid material and UV or visible irradiation to the generation of •OH- and •O2- radicals, which oxidize the MPs particles in water volumes. TiO2-based materials or semiconductors-based composites with low bandgap energy are commonly used as photocatalysts. Some limitations for the treatment of water samples with MPs of different sizes and complex conditions for the exposure to light should be surpassed. In addition, more efficient photocatalytic systems should be developed for faster and sustainability processes at low costs. Magnetic extraction is a technique that allows the removal of MPs by means of an external magnetic field that attracts MPs particles with Fe nanoparticles attached at their surface. Large-sized PP, PVC, PU, PS, and HDPE particles can be efficiently removed. The magnetic extraction is influenced by the size and surface area of the MPs, where better removal is observed for small particles. Magnetic extraction is a potential option for the MPs degradation, however, significant amounts of Fe nanoparticles for effective recovery are needed and designs for scaling this process for larger particle sizes are required. Ozonation technique allows degraded MPs in water or wastewater volumes using ozone with high efficiency, but full degradation is not reached using ozone. The reactive ozone molecules attack MPs particles and break down their chemical structure and MPs degradation is proportional to the amount of ozone used. Ozonation can be used as a pretreatment step to degrade the chemical structure of the MPs and later MPs particles are subsequently degraded by biological methods or some other. One negative aspect is the formation of harmful byproducts which complicates the large-scale water treatment.

Undoubtedly, the efforts made for the removal and degradation of microplastics in water are remarkable and continue to develop. For example, the proposal of biodegradable materials, to be used as adsorbents, catalysts or photocatalysts, for the elimination of microplastics in water matrices is quite promising, since it is aligned with the imperative of environmental sustainability. In this sense, the development of nanotechnology and biotechnology represents a significant advance in the search for effective methods for elimination of MPs. Therefore, it is necessary that the scientific and industrial community become the main source of solid proposals for the elimination of microplastics in water bodies and thus avoid the devastating consequences for all living beings on the planet.

## Data Availability

No datasets were generated or analysed during the current study.

## Abbreviations

EAP: East Asian and Pacific
FB: Fibre
FI: Film
FM: Microfoam
FR: Fragment
HA: Humic Acid
HM: Heavy Metal
MAP: Macroplastic
MBD: Microbead
ME: Mesoplastic
MFB: Microfibre
MFI: Microfilm
MFR: Microfragment
MMP: Mini-microplastics
MP: Microplastics
MSW: Municipal Solid Waste
NP: Nanoplastics
OM: Organic Matter
PCBs: Polychlorinated biphenyls
PE: Polyethylene
PFASs: Perfluorinated/Poly-fluorinated Alkyl Substances
PLT: Plastic
POM: Particulate Organic Matter
POPs: Persistent Organic Pollutants
PP: Polypropylene
PS: Polystyrene
PSMP: Polystyrene Microplastic
PT: Pellet
PTFE: Polytetrafluorethylene
TSS: Total Suspended Solids
UV: Ultraviolet
WWTP: Wastewater Treatment Plant
